# Exploring the interactions between *Nosema ceranae* infection and the honey bee gut microbiome

**DOI:** 10.1038/s41598-024-67796-y

**Published:** 2024-08-29

**Authors:** Edmund Lau, Jessica Maccaro, Quinn S McFrederick, James C. Nieh

**Affiliations:** 1 School of Biological Sciences, Department of Ecology, Behavior, and Evolution, University of California San Diego, La Jolla, CA 92093 USA; 2 Department of Entomology, University of California, Riverside, CA 92521 USA

**Keywords:** Microsporidia, *Nosema ceranae*, Microbiome and disease resistance, *Gilliamella*, Animal physiology, Microbial ecology

## Abstract

Managed colonies of the European honey bee, *Apis mellifera*, have faced considerable losses in recent years. A widespread contributing factor is a microsporidian pathogen, *Nosema ceranae,* which occurs worldwide, is increasingly resistant to antibiotic treatment, and can alter the host’s immune response and nutritional uptake. These obligate gut pathogens share their environment with a natural honey bee microbiome whose composition can affect pathogen resistance. We tested the effect of *N. ceranae* infection on this microbiome by feeding 5 day-old adult bees that had natural, fully developed microbiomes with live *N. ceranae* spores (40,000 per bee) or a sham inoculation, sterile 2.0 M sucrose solution. We caged and reared these bees in a controlled lab environment and tracked their mortality over 12 d, after which we dissected them, measured their infection levels (gut spore counts), and analyzed their microbiomes. Bees fed live spores had two-fold higher mortality by 12 d and 36.5-fold more spores per bee than controls. There were also strong colony effects on infection levels, and 9% of spore-inoculated bees had no spore counts at all (defined as fed-spores-but-not-infected). *Nosema ceranae* infection had significant but subtle effects on the gut microbiomes of experimentally infected bees, bees with different infection levels, and fed-spores-but-not-infected vs. bees with gut spores. Specific bacteria, including *Gilliamella* ASVs, were positively associated with infection, indicating that multiple strains of core gut microbes either facilitate or resist *N. ceranae* infection. Future studies on the interactions between bacterial, pathogen, and host genotypes would be illuminating.

## Introduction

Animal pollination services play a significant role in global food crop production, contributing about 35% of the total production^1,2^. About 75% of the world's food crops are at least partially dependent on insect pollination ^3^, with wild insects, including native bee species, making meaningful contributions^4^. However, commercial honey bees, such as the European honey bee *A. mellifera*, are crucial for the successful pollination of many crops. The estimated yearly value of commercial *A. mellifera* colonies as pollinators is between $12.3 and $16.4 billion in the U.S. alone^5^. The demand for commercial European honey bees as pollinators has increased as the proportion of pollinator-dependent crops in agriculture has tripled over the past 50 years^6^. Colony losses have also been observed globally to a degree that is not normal, pointing to poor overall health^7^. Contributing factors include migratory beekeeping, poor nutrition, infestations with *Varroa destructor*, and pathogens such as *Nosema ceranae*, which have received particular attention^8,9^. Interactions between these factors, such as pesticide exposure increasing susceptibility to *Varroa destructor* and *N. ceranae*, have proven problematic^8, 10^.

One particular pathogen, *N. ceranae*, has infected between 46–63% of honey bees sampled in different studies conducted around the world^11–14^. A new genus name was proposed for this pathogen, *Vairimorpha ceranae* ^15^, but we will use the name, *N. ceranae*, based upon more recent literature (Bartolome et al., 2024). This microsporidian originated from the Asian honey bee (*Apis cerana*) and has since been found to infect other honey bee species, including *Apis mellifera*, *Apis florea*, and *Apis dorsata*^16^. Within *A. mellifera*, the invasive *N. ceranae* has surpassed the endemic pathogen, *Nosema* a*pis*, in terms of distribution and infectivity ^17–19^. In France, some colonies have been found to contain eight times more *N. ceranae* than *N. apis*^20^, likely due to commercial trade in infected *A. mellifera* colonies^21^. Our research therefore focused on *N. ceranae*.

*Nosema ceranae* infection has multiple effects including suppressed immune response, digestive tissue degeneration, and premature mortality^22–24^. Infection can also modify pheromones essential for normal worker behavior, reduce brood rearing, and induce earlier foraging activity ^24–26^. These effects reduce colony fitness^27^, and *N. ceranae* can thus significantly decrease the survival of colonies in poor health, particularly in conjunction with other factors such as insecticide exposure^10,28,29^. In general, many of *N. ceranae’*s effects seem to manifest in the bee gut. Infected bees exhibit modified behaviors such as increased sucrose consumption and lower tendencies to share food^30^. Infected midgut cells also have a reduced ability to absorb nutrients^23^. Because *N. ceranae* exclusively infects the bee gut, it should have a strong effect on the gut microbiome^31–33^.

Globally, the microbiome of western honey bees has the same nine bacterial types^34,35^. This microbiome is colonized and matures approximately 5 d after adult emergence^36^. These microbes contain genes that are involved in host immune response, metabolism, growth, and development^35,37,38^. Honey bee specific microbes and *N. ceranae* co-occur abundantly in the midgut and hindgut ^36,39^. Researchers are increasingly focused on these interactions. Pollen patties inoculated with honey bee gut bacterium, *Parasaccharibacter apium,* lowered *Nosema* levels in workers that were challenged with *Nosema*^40^*.* Inoculation with *Bifidobacterium* and *Lactobacillus* strains isolated from the bee gut also reduced *N. ceranae* infection levels^41^. However, some studies have shown that an imbalance of microbiota composition (dysbiosis), induced via bacterial inoculation, can increase mortality and susceptibility to parasites such as *Nosema* ^35,42–45^. Understanding how *N. ceranae* infection alters the gut microbiome therefore has implications for developing treatments to combat the effects of infection and dysbiosis.

Feeding workers the same dose of live *N. ceranae* spores can lead to markedly different infection outcomes, with some bees having no spores at all in their gut while others can have infections exceeding one million gut spores^32,46,47^. Studies have identified significant variation based on colony identity, suggesting either that genetic background, the microbiome, or multiple factors may be involved^32,47^. We hypothesized that the gut microbiome may play an important factor in the ability of individual bees to resist *N. ceranae* infection. We, therefore, conducted a screening experiment in which we fed recently emerged honey bees with the same dose of live *N. ceranae* spores, divided the resulting bees into different groups based on their level of subsequent infection as measured by midgut spore counts, and analyzed their microbiomes. We note that changes in microbiome composition may reflect the outcome of infection, not initial conditions that helped bees resist infection. It is not possible to sample the microbiome of a living bee before and after infection. However, the information obtained after infection is still informative because we know relatively little about the association between *N. ceranae* infection and the bee microbiome.

## Materials and Methods

### Study site and colonies

We conducted our experiment on 15 *Apis mellifera ligustica* colonies at the Biology Field Station (32°53′07.9″N 117°13′55.1″W) apiary at the University of California San Diego. All colonies were healthy, based on standard inspection techniques^48^. Before being used for our experiments, we randomly sampled foragers (bees with pollen on their legs) returning to the colonies and dissected out 10 bees per colony to check for potential *N. ceranae* infections. None of the bees sampled had any gut spores.

### General methods

#### Collection of recently emerged bees

To obtain recently emerged bees, we first took out a frame from one of the colonies and located a large patch of capped worker brood. Using a brush, we carefully removed all the adult worker bees from the area and then used a corner of a hive tool to gently lift the caps off the worker broods to reveal the developing pupae. We selected pupae with deep purple eyes, which indicate they are close to eclosion. Then, we placed a sterile wire cage (5 × 7.5x2.5 cm) around the pupae and carefully pushed it into the wax to keep all sides even and tight. After putting the frame back in its original slot in the colony, we checked the cage every 24 h for adult emergence. Once the bees had emerged, they remained inside their colonies, confined within their wire cages and we waited 5 days to collect them, as honey bee workers need to develop their characteristic microbiome through food exchange and grooming with adult workers. After 5 days, we removed the frame, brushed off any other bees, and placed two sterile plastic cages (11.5 × 11.5x9 cm) on a table, facing upward with their sliding doors open. Holding the frame over the plastic cages with the wire cage closest to the opening, we removed the wire cage from the frame and quickly brushed the bees into the plastic cages. Then, we closed the cages with the sliding door.

#### Spore preparation and counting

We prepared fresh *N. ceranae* spores which consisted of standard extraction and purification of spores at room temperature from heavily infected bees less than 12 h before they were fed to bees^46^. We followed the standard procedure for measuring infection levels, using a hemocytometer to count the number of spores per bee^49^. We used PCR analysis to confirm that the spores were *N. ceranae* (see methods of^46^)*.*

#### Feeding recently emerged bees with *Nosema ceranae*

After obtaining the bees, we divided them into two groups: a control group (each bee received a 5 µl dose of sterile 2.0 M sucrose solution) and an experimental group (each bee received 40,000 spores in 5 µl of sterile 2.0 M sucrose solution, chosen because it effectively infects bees,^46^). To feed the bees, we placed them in individual, sterile vials and inserted a micropipette tip filled with the appropriate treatment into the vial lid. We positioned the vials in a tray beneath LED lights (SMD 3528, 240 lumens/m) to encourage feeding^32^. If the bees did not consume all of the solution within 30–60 min, we manually fed them the remaining solution to ensure that each bee received the same dose. After they finished feeding, we placed 25 bees in each cage, with all bees in the same cage receiving the same treatment. To keep the bees alive during the experiment, we placed a 5 ml syringe filled with approximately 3 ml of 2.0 M sterile sucrose solution in each plastic cage and returned the cages to the incubator.

#### Gut dissection and *Nosema ceranae* extraction

We maintained the bees in the cages inside a dark incubator set to the standard conditions of 34 °C and 70% relative humidity for caged adult worker bees^50^. Every 2 days, we monitored the bees, refilled their sucrose solution, recorded any mortality, and removed any dead bees.

After 12 days, chosen because the spores should fully mature by then (Fries et al., 2013), we placed all surviving bees in individual microcentrifuge tubes on ice for 10–15 min and then dissected them. For each bee, we swiftly removed the midgut and rectum, as the midgut is the main location of *N. ceranae*^39^ and the rectum holds the majority of the gut microbiome^36^. We placed the gut and rectum into a microcentrifuge tube containing 100 µl of bee gut extraction buffer, which was chilled and homogenized using a Kontes motorized pestle for 30 s. Each liter of bee gut extraction buffer, with a pH of 7.4, contained 1.45 mM NaCl, 0.02 g Peptone, and 500 µl Tween 20. The buffer was sterilized through autoclaving. We then transferred 30 µl of the homogenized gut solution to a separate microcentrifuge tube for microbiome analysis and preserved a part of the sample for future culturing by transferring another 30 µl of the original gut solution to a third tube with 30 µl of 30% reagent grade glycerol, which was then vortexed. To avoid contamination, for each bee, we used a different set of dissection tools, a different pestle, and a different microcentrifuge tube. Before use, all tools and pestles were thoroughly sanitized by washing with lab detergent, rinsed with 100% ethanol, rinsed multiple times with deionized water, and then autoclaved. All pipette tips were discarded after one use. All samples were kept on ice at all times and were stored at − 70 °C to prevent microbiome degradation.

#### Characterizing microbiota composition

The gut samples (midgut and hindgut) of bees from 10 randomly selected colonies (out of the 16 that we used) were collected and transferred to sterile 96-well sample extraction plates from the DNeasy extraction kit provided by Qiagen (Valencia, CA). To extract DNA, 50 μL of a mixture of 0.1 mm glass beads and two sterile 3.2 mm steel beads were added to each well. The samples were lysed by adding 180 μL of buffer ATL (Qiagen, Valencia, CA) to each well, then subjected to bead beating using a Tissue Lyser (Qiagen, Valencia, CA) for six minutes at 30 Hz. Twenty μL of proteinase K was added to each sample and incubated overnight at 56 °C. The extraction was completed following the protocol recommended by the DNeasy Blood and Tissue kit (Qiagen, Valencia, CA).

To determine the composition of the microbiota, we utilized established protocols to conduct 16S rRNA gene analysis. For the PCR reactions, we used the 799F (CMGGGTATCTAATCCKGTT) and 1115R (AGGGTTGCGCTCGTTG) primers that target the V5 and V6 regions of the 16S rRNA gene and excluded plastid regions^32,51,52^. The sequencing construct was built using a dual barcoding approach with two primer sets^53,54^. The PCR reactions were cleaned using the PureLink Pro PCR Clean-Up Kit (ThermoFisher Scientific, Waltham, MA), then each sample was normalized to an equal molarity using SequalPrep normalization plates (ThermoFisher Scientific, Waltham, MA) and finally, the reactions were pooled and sequenced with a 2 × 300 bp paired-end run using V3 reagents on the Illumina MiSeq platform.

#### Statistics and bioinformatic analyses

To determine the effects of treatment on spore levels, we used Analysis of Variance (ANOVA) with log-transformed spore counts (after inspecting model residuals) as our response variable and used colony identity, treatment, and the interaction colony x treatment as fixed effects. Cage name was a random effect nested within colony. Colony identity was a fixed effect because we explicitly wished to test the hypothesis that colonies varied. Although these data are counts, the measurement per bee ranged from 0 to over 41 million in increments of 5000 and therefore closely approximated a continuous variable suitable for ANOVA. We used JMP Pro v14.3.0.

To test if the proportion of surviving bees and treatment affected the mean spore count per bee in a cage, we used a Mixed Model (REML algorithm), calculated the proportion of surviving bees (number of living bees at the end of a trial/number of bees at the beginning of the trial), and tested if the proportion of surviving bees, treatment, and their interaction explained variation in the log-transformed average spore counts per bee. In this analysis, each cage was an individual data point, and colony was a random variable. We used JMP Pro v14.3.0.

To determine the effects of treatment (control bees that were not fed spores vs. experimental bees that were fed spores) upon survival, we first used a simple Kaplan–Meier survival model without colony, and then ran a Proportional Hazards Survival model with censoring and colony, treatment, and the interaction colony x treatment as fixed effects. We report model results as Effect Likelihood Ratio tests (L-R chi-square tests). We used JMP Pro v14.3.0 and reported a mean ± 1 standard error. To test the effects of treatment and average mortality per cage after 12 days on the log-transformed average spore counts per surviving bee per cage, we ran a Mixed Model (REML algorithm) with colony as a random effect and the interaction of average mortality per cage x treatment. When interactions were not significant, we eliminated them and reported the results of the reduced model.

To look at the influences of treatment on the microbiome, we processed the 16S rRNA gene data using QIIME2 version 2017–1149. We evaluated the quality scores of the DNA sequence and removed low-quality regions and chimeras, using the default parameters of DADA251. We then assigned taxonomy to each amplicon sequence variant (ASV) through two methods: (1) by training the Silva database (v. 12852) with our primer set in QIIME2 and using the sklearn classifier53 and (2) using NCBI’s 16S rRNA database to conduct local BLAST searches and pull out the taxonomy of the top hit, the top hit’s accession number, and the percent identity of the query to the top hit.

The statistical analyses were conducted with R version 4.03. We removed 18 contaminants that were present in our blanks with the R-package decontam (ver 1.10.0), using a conservative threshold of 0.5. We also filtered out mitochondria or chloroplasts in QIIME2. To normalize the number of sequences per library, we ran alpha-rarefaction in QIIME2 and selected 2500 reads per sample to retain most samples while still capturing the majority of the diversity of the samples (Fig. S1). This rarified feature table was then analyzed further with the R vegan package (ver 2.5–7).

**Figure 1:**
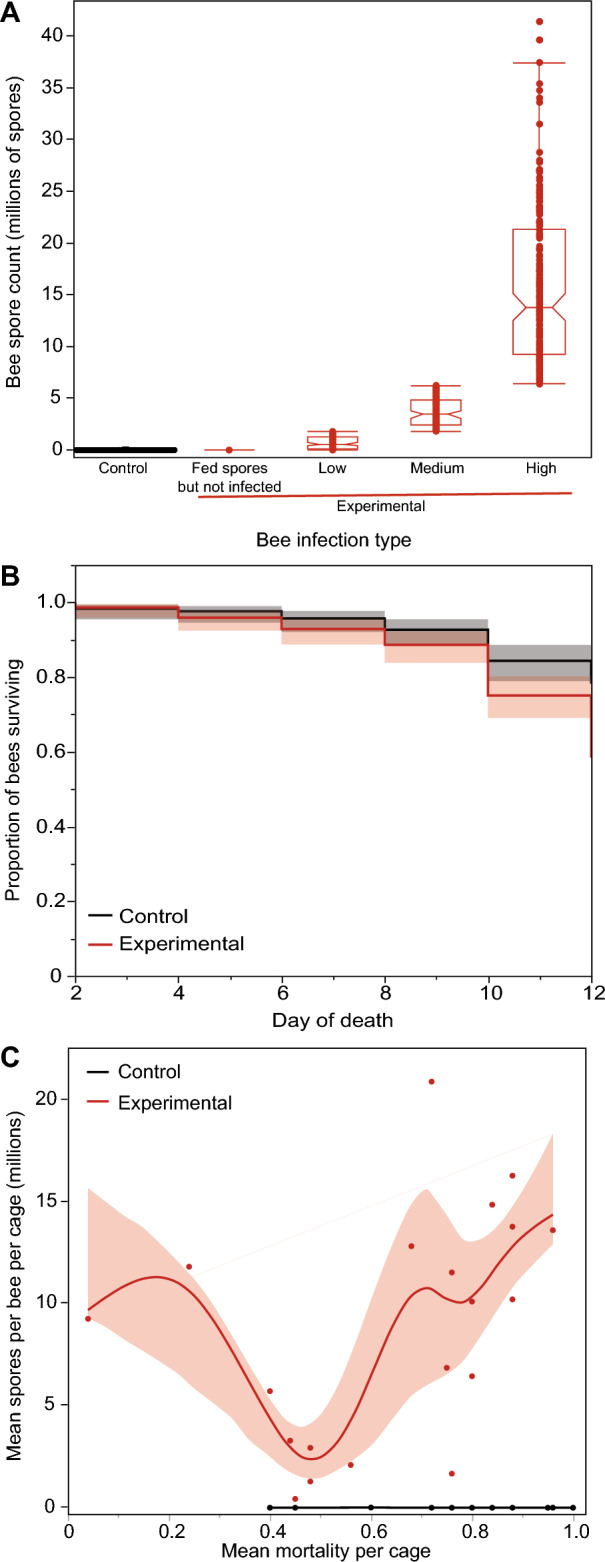
Spore counts and survival in control bees (fed no spores in sucrose solution) and experimental bees (fed spores in sucrose solution) after 12 days are shown. (**A**) *N. ceranae* infection levels were classified into four groups: fed-spores-but-not-infected bees with no spore counts and low, moderate, and high spore count bees (low=spore counts<median, moderate=median to 75th quartile, high=>75th quartile, see Table S1). (**B**) Survival plot showing that experimental bees had lower survival than control bees (see Results, *P* < 0.0001). (**C**) There is no relationship between mean mortality per cage and mean spores per bee per cage. However, experimental bees had significantly higher spore counts than control bees (spline lines and 95% confidence intervals shown as shaded areas).

We measured alpha and beta diversity to look at differences between (1) uninfected bees versus infected bees and (2) the level of infections divided into four factor levels based on quartiles of the spore load data in bees whose microbiomes we analyzed: control bees that were never fed spores and had zero spores (*control bees*), bees with spore counts below the median spore count value (*low spore count bees*), bees with spore count ranging from the median to the 75th quartile (*moderate spore count bees*), and bees with spores above the 75th quartile (*high spore count bees).* We based our quartile levels on bees whose microbiomes we analyzed (Table S1) because the main purpose of creating different infection levels was to understand our microbiome results in greater detail. However, for consistency, we applied these same quartiles to the infection data for all bees (Table S1, Fig. 1).

We also compared (3) bees that were given the sham treatment (*control bees* fed only pure sucrose without spores) and bees that were fed spores but subsequently had no gut spores (*fed-spores-but-not-infected bees*). To evaluate beta diversity, we created Bray–Curtis dissimilarities matrices and performed nonmetric multidimensional scaling using metamds in vegan. We used vegan betadisp to test for homogeneity of multivariate dispersions and treated colony identity as a block. The results were visualized with ordiellipse, where the ellipses represent 95% confidence intervals.

To look at alpha diversity we ran two linear mixed-effect models using the lme4 package in R (ver. 1.1.27.1) and one linear model. The response variable for all three models was the number of ASVs in the bees and the predictor variable was the treatments described above. For our analyses of (1) control versus treatment and (2) levels of infection, we used colony and cage numbers as random effects and ran linear mixed effects models. We could not use random effects in our (3) resistance model because it violated model assumptions, so we ran a simple linear model. Model assumptions were tested with the DHARMa package in R (ver 0.4.3). To determine the statistical significance of the two linear mixed effects models, we conducted Type II Wald chi-square tests. We used ancom in QIIME2 to test for ASVs that were differentially abundant between the microbiomes of the treatment groups. We used vegan’s betadisper function to compare dispersion between groups. Lastly, we used the phyloseq package in R for bacterial profiling/plotting the relative abundance of bacterial taxa.

## Results

### Effects of treatment on survival and spore counts

The outcome of being fed live spores varied among the bees, depending on their colony background (Fig. 1A). The highest spore count, 12 days after bees were fed live spores, was 41,400,000. Out of the 506 control bees that were not fed any spores, only 8% had any spores (22,024 ± 20,063 spores/bee in bees with any spores). Out of the 497 bees that were fed live *N. ceranae* spores, only 9% were not infected (0 spore counts, defined as “fed-spores-but-not-infected”).

Our model accounted for 83% of the variance in spore count. There were significant differences between the treatment groups (control bees that were not fed spores vs. experimental bees that were fed spores: *F*_1,37_ = 464.18, *P* < 0.0001) and colonies (*F*_15,38_ = 2.20, *P* = 0.026), and no significant treatment x colony interaction (although this interaction was close to significant, *F*_15,23_ = 2.06, *P* = 0.06). Cage was also a significant effect (Wald *P* value = 0.0001) and accounted for 30% of model variance.

Out of the 15 colonies, 42.9% of bees fed *N. ceranae* spores died prior to day 12, while only 22.5% of control bees died in the same time frame. In the simple survival model that only tested the effects of treatment on survival, bees fed spores had significantly lower survival than bees fed sucrose only (L-R Chi-square = 46.24, 1 df, *P* < 0.0001, Fig. 1B). However, in the Proportional Hazards model that included colony identity as a factor, survival depended upon colony background. This model showed no significant differences in treatment (L-R Chi-square = 2.90 × 10^–6^, 1 df, *P* = 0.9986), because survival strongly depended on source colony (L-R Chi-square = 122.54, 14 df, *P* < 0.0001), as shown by the significant interaction between colony and treatment (L-R Chi-square = 31.90, 14 df, *P* = 0.004). For seven colonies, the mortality rate was significantly higher in the experimental bees compared to the control bees (L-R Chi-square ≥ 4.13, 1 df, *P* ≤ 0.04), while in the remaining eight colonies, there were no significant differences in mortality between the two treatments (L-R Chi-square ≤ 3.50, 1 df, *P* ≥ 0.06). In the eight colonies where feeding bees spores significantly reduced their survival, the experimental survival proportion at day 12 was 0.45 ± 0.03 (mean ± 1 SE). In contrast, in the seven colonies where survival was not significantly reduced, the experimental survival proportion at day 12 was 0.64 ± 0.02.

We also tested if average survival per cage and treatment could predict the average spore count per bee per cage. Our model accounted for 76% of the variance in spore counts. Treatment significantly predicted the average spore count per bee per cage (*F*_1,33_ = 149.80, *P* < 0.0001), and there was a significant effect of survival (*F*_1,10_ = 5.80, *P* = 0.038, Fig. 1C). The interaction between average survival and treatment (*F*_1,35_ = 2.93, *P* = 0.10) was not significant. Colony (a random effect) accounted for < 1% of the variance in this model. Essentially, control bees had almost no spores, and experimental bees were highly infected such that cages in which a higher proportion of bees survived during the 12-day trial had a higher average spore count.

### Effects of spore treatment on microbiome composition

**Figure 2:**
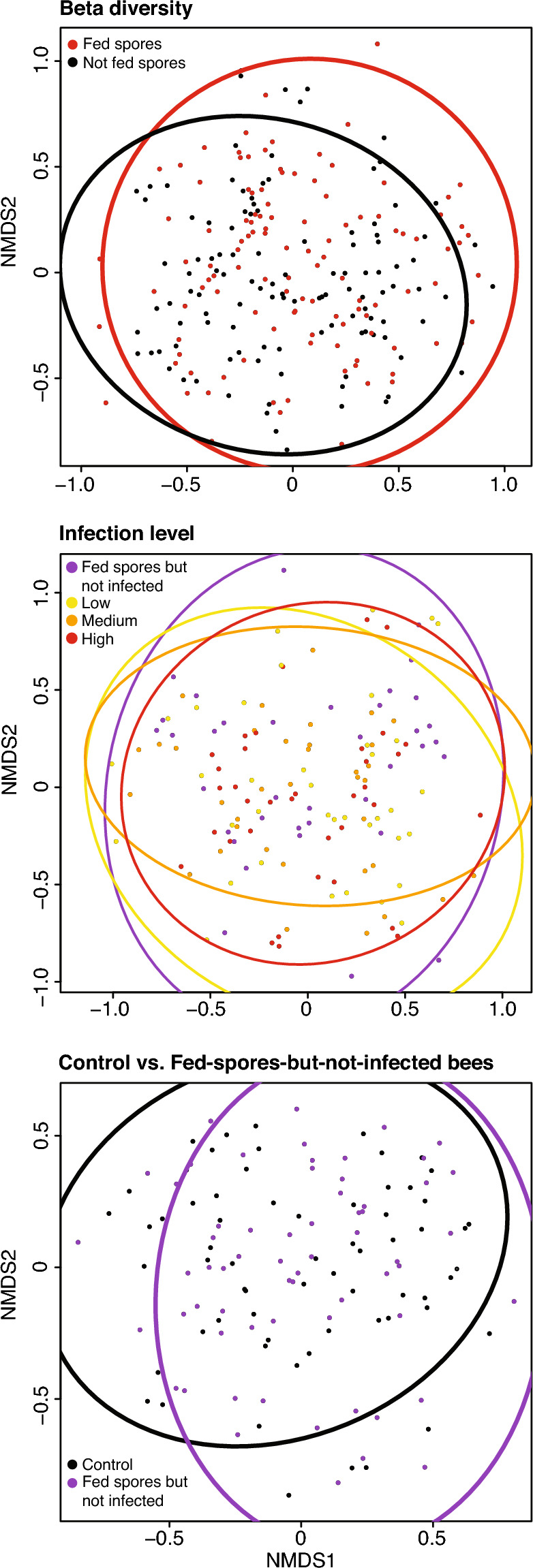
Microbial community compositions are shown. (**A**) Control bees were fed no spores, only the sucrose solution, and experimental bees were each fed 40,000 spores in sucrose solution. (**B**) *N. ceranae* infection levels were classified into four groups: fed-spores-but-not-infected bees with no spore counts and low, moderate, and high spore count bees (based upon quartiles, see Table S1). (**C**) Both control and fed-spores-but-not-infected bees had no spore counts, but beta diversity (*P* = 0.002) and beta dispersion (*P* = 0.001) were significantly different between these two groups of bees. The ellipses show 95% confidence intervals per group.

The beta diversity between the microbiomes of treatment and control bees was significantly different (*F*_1,228_ = 4.43, *P* < 0.001, *R*^2^ = 0.019). The overall beta dispersion between treatment and control bees was not significant (*F*_1,228_ = 6.27, *P* = 0.13). However, beta dispersion of the microbiome was significantly different between the different infection groups (*F*_3,142_ = 3.61, *P* = 0.048). Pairwise comparisons of beta dispersion for the different groups revealed a significant difference between the fed-spores-but-not-infected bees and the high spore count bees (*P* = 0.05) and significant differences between the median spore count bees and fed-spores-but-not-infected vs. median spore count bees (*P* = 0.002) and low vs. median spore count bees (*P* = 0.028, Fig. 2).

Concerning infection levels, beta diversity was significantly different between the infection groups (*F*_3,142_ = l 0.99, *P* = 0.03, *R*^2^ = 0.04). In pairwise comparisons, all of the groups differed significantly from each other except between the low spore count and fed-spores-but-not-infected bees (no infection) and between moderate spore count and high spore count bees. There were no significant differences in alpha diversity between the different infection level groups (*Χ *^2^_3_ = 3.29, *N* = 97,155, *P* = 0.07). Three uncultured gamma proteobacteria (*Gilliamella*) ASVs and one unknown bacteria ASV had statistically significant differential abundance between the infection levels (and most abundant in the high infection group, Table S1). A pairwise BLAST search revealed that the *Gilliamella* ASV that was significantly more abundant in high spore count bees differed from the two *Gilliamella* ASVs that positively correlated with infection in our previous work^32^.

**Figure 3:**
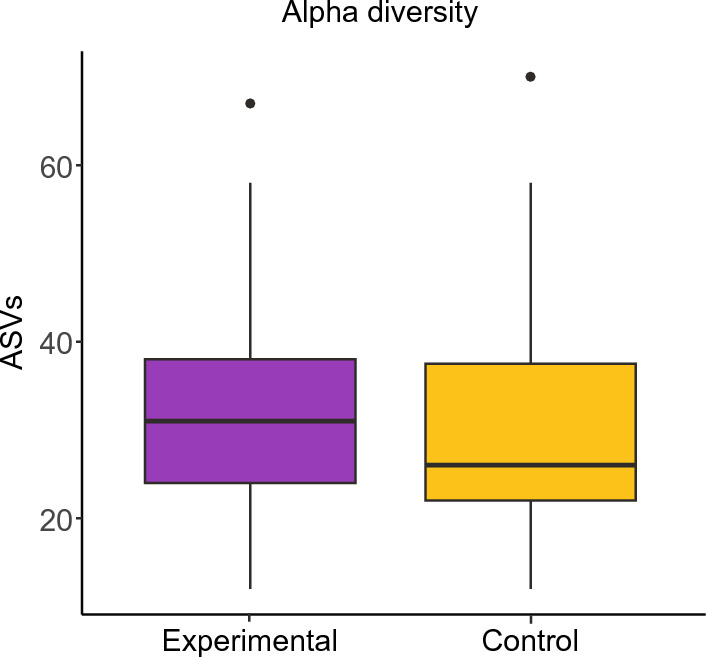
Alpha diversity is shown. There were significant differences in alpha diversity between the control bees (bees not fed spores) and the experimental bees (bees that were fed live spores, *Χ *^2^_1_ = 4.97, *N* = 176, *P* = 0.026).

Feeding bees live spores significantly affected alpha diversity as compared to uninfected control bees *(Χ* ^2^_1_ = 4.97, *N* = 176, *P* = 0.026, Fig. 3). There were statistically significant differences in the abundance of five uncultured *Lactobacillus* (a higher abundance in the treatment for three out of the five *Lactobacillus*) and one *Bombella* (higher abundance in the control) (Table S1).

Finally, with respect to resistance, beta diversity was significantly different between fed-spores-but-not-infected and control bees (*F*_1,139_ = 2.95, *P* < 0.001, *R*^2^ = 0.02). Beta dispersion was significantly different between control and fed-spores-but-not-infected bees (*F*_1,126_ = 13.58, *P* = 0.001). However, there were no significant differences in alpha diversity between control and fed-spores-but-not-infected bees (*Χ* ^2^_1_ = 0.0484, *N* = 100, *P* = 0.826). Two uncultured *Lactobacillus* sp. ASVs were statistically significantly differentially abundant between control and fed-spores-but-not-infected bees (one was more abundant in fed-spores-but-not-infected bees while the other was more abundant in control, Table S1). Besides the few taxa mentioned, there were no significant differences in the relative abundance of bacteria between groups. We also plotted the relative abundance of the top ten bacterial species and genera by control (uninfected bees), bees fed-spores-but-not-infected, low infection, medium infection, and high infection levels in Fig. S2.

## Discussion

We aimed to determine the impact of feeding live spores on the survival and infection levels of bees in a controlled laboratory setting. Our prior *N. ceranae* experiment involved returning bees fed spores back to their colonies. However, this treatment resulted in low levels of infection among the control bees, likely due to the interaction of infected and control bees^32^. To overcome this limitation, we isolated the control and experimental bees in separate cages in this experiment.

Feeding live *N. ceranae* spores to bees led to a significant increase in gut spore levels and a decrease in bee survival, as anticipated. Notably, infection levels varied significantly among different colonies, suggesting that factors such as the colony's genetic background, microbiome composition, or a combination of both, play a role in their resistance or susceptibility to *N. ceranae*. Bees exhibited subtle differences in their microbiome compositions that depended upon whether they were infected or sham inoculated, had different infection levels, or displayed resistance or susceptibility to infection. These shifts manifested as subtle changes in community composition (Beta-diversity as tested with PerMANOVA) but not differences in how variable communities were by treatment (Beta-dispersion). Alpha diversity (the number of bacterial taxa, in this case ASVs, per sample) also varied by treatment, but not in the presence or absence of core bacteria. Our findings therefore suggest that shifts in the abundance of core honey bee bacteria, rather than the presence or absence of specific bacteria, are associated with resistance to and the susceptibility of bees to different levels of infection with *N. ceranae.*

Multiple studies have shown that *N. ceranae* infection has a harmful effect on *A. mellifera*, so it is not surprising that infection reduces worker lifespan. Our bees fed with *N. ceranae* spores had a mortality rate of 43% after 12 days post-inoculation (dpi), which was significantly higher than the control bees' mortality rate of 23%. These results are similar to those reported in other studies, such as Goblirsch et al.^55^ and Milbrath et al.^47^.

Our treatment method for bees differed from previous studies in several ways, which may explain some of the differences in results. For example, our bees were only fed with a sterile sucrose solution, while some other studies provided their caged bees with non-sterile pollen and dietary supplements. We also caged our bees on comb in the colony for the first 5 days post-eclosion to allow them to obtain the colony microbiome.

We found that the bees fed with live spores were significantly more infected than control bees. On average, bees that were fed 40,000 live spores had infection levels of 7.7 million spores per bee at 12 dpi, with 91.5% of them being infected (defined as having at least one spore by microscopic analysis). This is in line with previous studies that showed that older bees, especially foragers, are the most susceptible and highly infected by *N. ceranae.*

Overall, there is considerable variation in infection levels among different experiments, which may be due to differences in *N. ceranae* purification and feeding techniques, bee age, and colony background^56,57^. Our study recorded the highest infection level to be 41.4 million spores per bee, which is comparable to levels found in other studies^56,57^. Further research is necessary to fully understand the mechanisms behind *N. ceranae* resistance and susceptibility in bees.

*Nosema ceranae* infection had a significant but subtle effect on honey bee gut microbiome composition. Bees that were either experimentally infected or sham inoculated showed subtle differences in the composition of their microbiomes. Similarly, bees that were fed-spores-but-not-infected or were fed spores and became infected also had some differences in their microbiomes. These results reflect differences in the abundances of ‘core’ honey bee bacteria instead of differences in presence or absence of specific bacteria. For example, a *Lactobacillus apis* ASV was at greater abundance in infected bees compared to control bees, while a *L. bombicola* ASV and two *Snodgrasella* ASVs were at lower abundance in infected bees. Previous work has shown that *Lactobacillus* spp. can mitigate *Nosema* infection either when administered alone^58^ or in conjunction with *Bifidobacterium* (Baffoni et al., 2015). Likewise, *Snodgrasella*, inhibits *Nosema* infection via host-immune priming and subsequent disruption of the pathogen’s redox system^59^. However, as noted by Raymann and Moran^35^, whether these correlations are driven by infection or are drivers of infection remains to be seen. While other factors account for the majority of microbiome variation, our finding that *N. ceranae* infection has subtle effects on honey bee gut microbiome composition is consistent with our previous study^32^, as well as the work of others^33,60–62^.

As in our previous research^32^, we found two *Gilliamella* ASVs that were positively associated with *N. ceranae* infection. However, these *Gilliamella* ASVs differed between our two studies. This result suggests that several or even many *Gilliamella* strains may either facilitate or be involved in resistance to *N. ceranae* infection, while other strains appear to not be affected or involved. Exploring strain-level interactions between gut microbes, gut pathogens, and hosts could be a rich future research direction.

Several other studies have demonstrated a positive correlation between certain microbiome bacteria and bee diseases. Schwarz et al.^44^ found that the presence of *Gilliamella apicola* increased in bees affected by the parasite, *L. passim*. Furthermore, stressed bees that were treated with *S. alvi* and *L. passim* had the largest amount of *G. apicola*. An analysis of bee colonies diagnosed with Colony Collapse Disorder (CCD), a rapid decline of honey bee colonies, displayed a consistent pattern of heightened *Gammaproteobacteria* (including *G. apicola* and *Frischella perrara*)^44^. Rubanov et al.^32^, showed that two specific sequence variants of *Gilliamella*, a core gut symbiont previously linked to gut dysbiosis, were significantly more prevalent in bees from colonies with high levels of *N. ceranae* as compared to those with low levels. However, Ye et al.^63^ discovered that the relative abundance of *Gilliamella* spp. was significantly reduced in bees infected with American foulbrood (AFB, caused by *Paenibacillus larvae*) or chalkbrood (caused by *Ascosphaera apis*). Additionally, Erban et al.^64^ found that *Citrobacter freundii* and *Hafnia alvei* were more abundant in association with AFB. Zhang et al.^33^ showed that *Bifidobacterium* spp. significantly increased with *N. ceranae* infection. Although the specific causes and mechanisms of this association are still unknown, these results suggest that indigenous *Bifidobacterium* spp. in honey bee hindguts may have no preventative effects on *N. ceranae* disease.

There is evidence that some bacteria can be associated with the ability of bees to resist disease. Erban et al.^64^ found that the bacteria *Enterococcus faecalis*, *Klebsiella oxytoca*, *Spiroplasma melliferum*, and *Morganella morganii* were more abundant in colonies that were either outside the zone in which AFB is found or within the AFB zone but asymptomatic. Daisley et al.^65^ reported that the use of probiotic lactobacilli improved colony resistance to AFB. Laboratory experiments with honey bee larvae showed that *Lactobacillus plantarum* Lp39, *Lactobacillus rhamnosus* GR-1, and *Lactobacillus kunkeei* BR-1 could reduce pathogen loads, increase expression of key immune genes, and improve survival during *P. larvae* infection. Borges et al.^66^ showed that feeding bees *Enterococcus faecium* reduced *N. ceranae* spore numbers without affecting bee mortality. Ye et al.^63^ showed that healthy larvae were significantly enriched in the bacterial genera *Lactobacillus* and *Stenotrophomonas*, as well as the fungal genera *Alternaria* and *Aspergillus*. The authors suggest that this enrichment of microorganisms may protect larvae from potential infections. In contrast, the relative abundance of *Gilliamella* spp. was significantly reduced in infected foraging bees. Finally, some bacteria have shown no impact on bee disease levels. Floyd et al.^67^ found no effect of *Parasacharribacter apium* strain C6 (now *Bombella apis*^68^) on European foulbrood (EFB), contrary to prior findings. Stephan et al.^69^found no effect of lactic acid bacteria dietary supplementation against AFB disease.

Some studies indicate that a more diverse microbiome may not necessarily be beneficial for honey bees. Zhang et al.^70^ found that bees fed a prebiotic had slightly higher pathogen counts but also lower mortality rates. Analysis of the bee microbiota suggested that infected bees had a similar composition to those with a longer lifespan, and the prebiotic seemed to enhance these similarities. Erban et al.^64^ showed that bees infected with AFB had microbiomes with higher alpha diversity than control bees. Napflin and Schmid-Hempel^71^ found that, in *Bombus terrestris*, higher microbiota OTU diversity was associated with lower resistance to *Crithidia bombi*. Parasite infection success can depend on microbiota composition, but the key alterations are elusive. The microbiota-host interaction before parasite exposure, rather than the exposure to the parasite itself, may be key.

In contrast, other studies suggest that having a diverse microbiome is beneficial. Mockler et al.^72^ found that high microbiome diversity was associated with lower levels of *Crithidia* infection in *Bombus impatiens*, while Harris et al.^73^ found that the complete community of gut bacteria is necessary to protect against the bacterial pathogen *Paenibacillius larvae.* It is important to consider that these results may vary depending on the species of bee, the pathogen in question, and the methods used to study the relationship between the microbiome and bee health. For example, *Nosema* is restricted to adult gut tissues, whereas other diseases affect immature stages and may have more systemic effects on the microbial community external to the gut. Finally, Li et al.^62^ inoculated bees with *N. ceranae* and fed them an antibiotic that eliminated their microbiomes. Eliminating the microbiome harmed bee immune functioning and made bees more susceptible to *N. ceranae* infection. Further research is clearly needed to fully understand the microbiome's role in honeybees' health.

Our findings therefore support the growing body of literature that highlights the correlation between specific microbiome bacteria and honey bee diseases. Interestingly, our results emphasize the changes in the abundance of core honey bee bacteria rather than the presence or absence of specific bacteria may be important for honey bee health.

Finally, we should consider that the microbiome may vary seasonally. Rouze et al.^74^ showed that exposure to the parasite *N. ceranae* and fipronil treatment can alter the abundance of certain bacterial species in the bee gut, potentially negatively impacting bee health. Almeida et al.^75^ found that the strongest determinant of honeybee microbiome composition was time, with clustering of the microbiome by time point observed across all apiaries. The study also found a correlation between the forager bee microbiome and hive health, as measured by the number of larvae, bees, and honey production. These findings highlight the importance of considering seasonal variation and the potential impact of environmental factors, such as forage availability, when studying the honey bee microbiome.

### Supplementary Information

Supplementary Information 1.

Supplementary Information 2.

## Data Availability

The datasets generated and/or analyzed during the current study are available in the Zenodo.com repository, at this DOI:10.5281/zenodo.10795522.
