# Supplementary material for: Exploring the interactions between Nosema ceranae infection and the honey bee gut microbiome
Source: Sci Rep. 2024 Aug 29;14:20037. doi: 10.1038/s41598-024-67796-y (PMC11358482; doi:10.1038/s41598-024-67796-y)
Supplement: Supplementary file 1 — Supplementary Information 1. [file 41598_2024_67796_MOESM1_ESM.pdf]

Diversity Statistics for "Exploring the interactions between Vairimorpha ceranae infection and the honey bee gut microbiome"

Edmund Lau, Jessica Maccaro, Quinn McFrederick, and James C. Nieh

5/10/2023

Load/Install packages with versions

```
version
```

```
##                _
## platform       x86_64-apple-darwin17.0
## arch           x86_64
## os             darwin17.0
## system         x86_64, darwin17.0
## status
## major          4
## minor          2.2
## year           2022
## month          10
## day            31
## svn rev        83211
## language       R
## version.string R version 4.2.2 (2022-10-31)
## nickname       Innocent and Trusting
```

```
if(!requireNamespace("BiocManager")){
  install.packages("BiocManager")
}
```

```
## Loading required namespace: BiocManager
```

```
#BiocManager::install("phyloseq")
library(phyloseq); packageVersion("phyloseq")
```

```
## [1] '1.42.0'
```

```
# '1.34.0'
library(ggplot2); packageVersion("ggplot2")
```

```
## [1] '3.4.2'
```

```
# '3.3.3'n

if (!requireNamespace("BiocManager", quietly = TRUE))
  cinstall.packages("BiocManager")
#install.packages("remotes")
#remotes::install_github("GuillemSalazar/EcolUtils")
#install.packages("spaa")
library(EcolUtils)
#BiocManager::install("decontam")
library(decontam)
packageVersion("decontam")
```

```
## [1] '1.18.0'
```

```
library(vegan)
```

```
## Loading required package: permute

## Loading required package: lattice

## This is vegan 2.6-4
```

```
packageVersion("vegan")
```

```
## [1] '2.6.4'
```

```
library(phyloseq)
#update.packages("phyloseq")
packageVersion("phyloseq")
```

```
## [1] '1.42.0'
```

```
library(dplyr)
```

```
##
## Attaching package: 'dplyr'

## The following objects are masked from 'package:stats':
##
##     filter, lag

## The following objects are masked from 'package:base':
##
##     intersect, setdiff, setequal, union
```

```
packageVersion("dplyr")
```

```
## [1] '1.1.2'
```

```r
library(tidyverse)
```

```
## -- Attaching core tidyverse packages ------------------------ tidyverse 2.0.0 --
## v forcats   1.0.0     v stringr   1.5.0
## v lubridate 1.9.2     v tibble    3.2.1
## v purrr     1.0.1     v tidyr     1.3.0
## v readr     2.1.4
```

```
## -- Conflicts ------------------------------------------ tidyverse_conflicts() --
## x dplyr::filter() masks stats::filter()
## x dplyr::lag()    masks stats::lag()
## i Use the conflicted package (<http://conflicted.r-lib.org/>) to force all conflicts to become errors
```

```r
packageVersion("tidyverse")
```

```
## [1] '2.0.0'
```

```r
library(lme4)
```

```
## Loading required package: Matrix
##
## Attaching package: 'Matrix'
##
## The following objects are masked from 'package:tidyr':
##
##     expand, pack, unpack
```

```r
packageVersion("lme4")
```

```
## [1] '1.1.33'
```

```r
library(DHARMa)
```

```
## This is DHARMa 0.4.6. For overview type '?DHARMa'. For recent changes, type news(package = 'DHARMa')
```

```r
packageVersion("DHARma")
```

```
## [1] '0.4.6'
```

```r
library(car)
```

```
## Loading required package: carData
##
## Attaching package: 'car'
##
## The following object is masked from 'package:purrr':
##
##     some
##
## The following object is masked from 'package:dplyr':
##
##     recode
```

```
packageVersion("car")
```

```
## [1] '3.1.2'
```

```
library(ggplot2)
packageVersion("ggplot2")
```

```
## [1] '3.4.2'
```

```
library(emmeans)
packageVersion("emmeans")
```

```
## [1] '1.8.5'
```

```
library(reshape2)
```

```
##
## Attaching package: 'reshape2'
##
## The following object is masked from 'package:tidyr':
##
##     smiths
```

```
packageVersion("reshape2")
```

```
## [1] '1.4.4'
```

```
library(RColorBrewer)
packageVersion("RcolorBrewer")
```

```
## [1] '1.1.3'
```

```
library(reshape2)
packageVersion("reshape2")
```

```
## [1] '1.4.4'
```

Decontam Code

This is to bioinformatically decontaminate our samples using contaminants in the blanks. Decontam tutorial http://127.0.0.1:31267/library/decontam/doc/decontam_intro.html follow this tutorial for phyloseqizing https://joey711.github.io/phyloseq/import-data.html#_mg-rast Here is their paper for decontam https://microbiomejournal.biomedcentral.com/articles/10.1186/s40168-018-0605-2

```
# I had to Phyloseq-ize my data
#Loaded my mapping files with this code after adding a col for sample and control
map <- readxl::read_xlsx("phyloseq_master.xlsx", sheet = "sample_map")
```

```
## New names:
## * `` -> `...4`
```

```r
samples_df <- map %>%
  tibble::column_to_rownames("SampleID")

#ASV table
feat_tab_ID <-readxl::read_xlsx("phyloseq_master.xlsx", sheet = "feature_table")
otu_mat <- feat_tab_ID %>%
  tibble::column_to_rownames("ASV_ID")

# Tax table in excel I had to add the column names "Phylum-species" and separate them because the qiime
tax <-readxl::read_xlsx("phyloseq_master.xlsx", sheet = "taxonomy_tab_clean")
tax_mat <- tax %>%
  tibble::column_to_rownames("ASV_ID")

#Make Matrix
otu_mat <- as.matrix(otu_mat)
tax_mat <- as.matrix(tax_mat)

#Phyloseq-ize
OTU <- otu_table(otu_mat, taxa_are_rows = TRUE)
TAX = tax_table(tax_mat)
samples = sample_data(samples_df)
ps <- phyloseq(OTU, TAX, samples)

# Now time to actually move through the tutorial in the prevalence section.

# Make phyloseq object of presence-absence in negative controls and true samples first just to see how
ps.pa <- transform_sample_counts(ps, function(abund) 1*(abund>0))
ps.pa.neg <- prune_samples(sample_data(ps.pa)$SampleOrControl == "Control", ps.pa)
ps.pa.pos <- prune_samples(sample_data(ps.pa)$SampleOrControl == "Sample", ps.pa)


# More "aggresive" threshold=0.5, that will identify as contaminants all sequences thare are more preva
sample_data(ps)$is.neg <- sample_data(ps)$SampleOrControl == "Control"
contamdf.prev <- isContaminant(ps, method="prevalence", neg="is.neg")
head(which(contamdf.prev$contaminant))
```

```
## [1] 127 213 486 493 495 541
```

```r
contamdf.prev05 <- isContaminant(ps, method="prevalence", neg="is.neg", threshold=0.5)
table(contamdf.prev05$contaminant)
```

```
##
## FALSE  TRUE
##   695    18
```

```r
write.table(contamdf.prev05, file = "decomtam0.5.txt", sep = "\t", quote = FALSE, row.names = T)

# Make data.frame of prevalence in positive and negative samples
df.pa <- data.frame(pa.pos=taxa_sums(ps.pa.pos), pa.neg=taxa_sums(ps.pa.neg),
                    contaminant=contamdf.prev$contaminant)
ggplot(data=df.pa, aes(x=pa.neg, y=pa.pos, color=contaminant)) + geom_point() +
  xlab("Prevalence (Negative Controls)") + ylab("Prevalence (True Samples)")
```

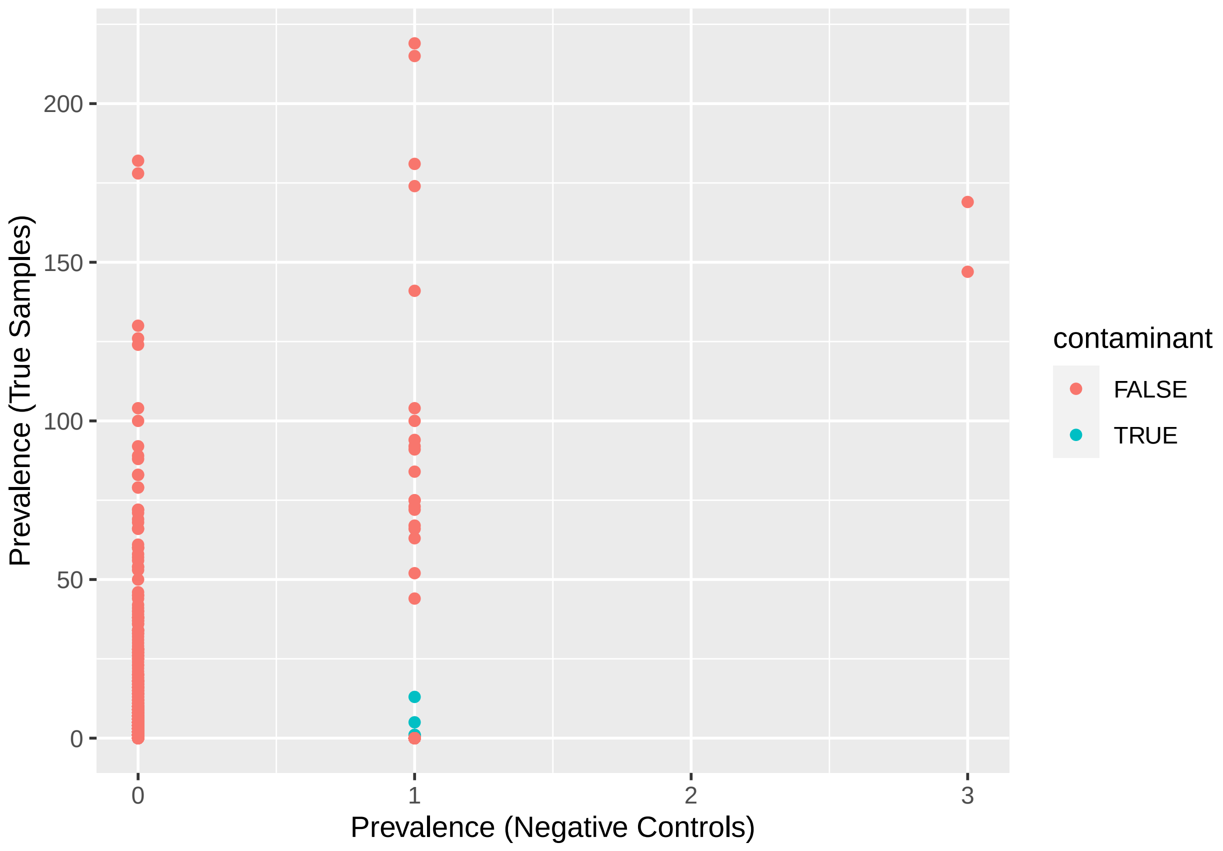

Beta Diversity Stats

Treatment Versus Control Load Files

I'm filtering out the controls that had infection from the OTU table using qiime2 and the mapping file.

Transpose the OTU table so that the ASVs were the columns and the sampleIDs were the rows. I also had to make sure to remove the '#' that is in the OTU header so R ould read the header. I also removed the samples from the metadata that didn't make it into the OTU table after rarifaction.

NMDS Stress

```
BC.m <- metaMDS(OTU.m, distance="bray", k=3,  trymax=1000)
```

```
## Square root transformation
## Wisconsin double standardization
## Run 0 stress 0.1888327
## Run 1 stress 0.1888328
## ... Procrustes: rmse 0.000267948  max resid 0.002874861
## ... Similar to previous best
## Run 2 stress 0.1906865
## Run 3 stress 0.1888326
## ... New best solution
## ... Procrustes: rmse 0.0002060943  max resid 0.002234397
## ... Similar to previous best
## Run 4 stress 0.1888331
```

```
## ... Procrustes: rmse 0.0001484706  max resid 0.001230508
## ... Similar to previous best
## Run 5 stress 0.1891309
## ... Procrustes: rmse 0.007671301  max resid 0.1081572
## Run 6 stress 0.188833
## ... Procrustes: rmse 0.0001146593  max resid 0.001158879
## ... Similar to previous best
## Run 7 stress 0.188833
## ... Procrustes: rmse 0.0003616888  max resid 0.004839256
## ... Similar to previous best
## Run 8 stress 0.1915923
## Run 9 stress 0.1888326
## ... New best solution
## ... Procrustes: rmse 0.0002512185  max resid 0.001972347
## ... Similar to previous best
## Run 10 stress 0.1899915
## Run 11 stress 0.1888343
## ... Procrustes: rmse 0.0005358564  max resid 0.004926457
## ... Similar to previous best
## Run 12 stress 0.1888333
## ... Procrustes: rmse 0.0003997135  max resid 0.003481285
## ... Similar to previous best
## Run 13 stress 0.1891307
## ... Procrustes: rmse 0.007562652  max resid 0.1078548
## Run 14 stress 0.1906906
## Run 15 stress 0.1891305
## ... Procrustes: rmse 0.007568734  max resid 0.1078872
## Run 16 stress 0.1895418
## Run 17 stress 0.1888332
## ... Procrustes: rmse 0.0001153681  max resid 0.0009655955
## ... Similar to previous best
## Run 18 stress 0.1888326
## ... Procrustes: rmse 0.0002017532  max resid 0.002094499
## ... Similar to previous best
## Run 19 stress 0.1898198
## Run 20 stress 0.1888325
## ... New best solution
## ... Procrustes: rmse 0.0002398339  max resid 0.002742319
## ... Similar to previous best
## *** Best solution repeated 1 times
```

```
stressplot(BC.m)
```

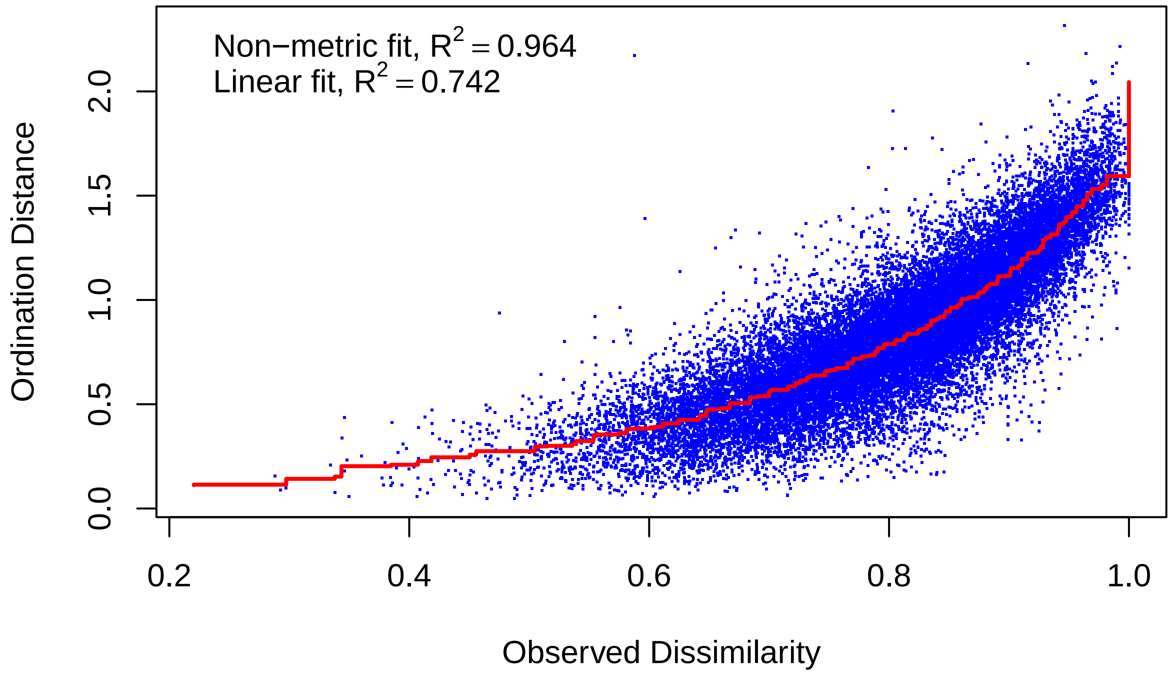


```
BC.m
```

```
##
## Call:
## metaMDS(comm = OTU.m, distance = "bray", k = 3, trymax = 1000)
##
## global Multidimensional Scaling using monoMDS
##
## Data:     wisconsin(sqrt(OTU.m))
## Distance: bray
##
## Dimensions: 3
## Stress:     0.1888325
## Stress type 1, weak ties
## Best solution was repeated 1 time in 20 tries
## The best solution was from try 20 (random start)
## Scaling: centring, PC rotation, halfchange scaling
## Species: expanded scores based on 'wisconsin(sqrt(OTU.m))'
```

After checking a few different k (from 3-5). They all had stress s < 0.2. So we went with k=3.

NMDS Plot Treatment V Control

```
setEPS()
postscript("FINAL_NMDS_plot_treatvcont.eps", horizontal = FALSE, onefile = FALSE, paper = "special", he
```

```
map$Treatment_factor<- factor(map$Treatment,levels = c("Exp", "Cont"))

plot(BC.m, "sites",
     main ="Microbial Community Composition by Treatment")
with(map,
     points(BC.m,
            display = "sites",
            pch = 20,
            col = c("red","black")))

legend("bottomright", legend=c("Experimental","Control"), col=c("red","black"), pch=20, cex = 0.75)

ordiellipse(
  BC.m,
  map$Treatment_factor,
  display = "sites",
  conf = 0.95,
  col = c("red","black"),
  lwd = 2.5
)


dev.off()
```

```
## pdf
##   2
```

Levels of Infection This section looks at whether there are differences in the level of infection split into 4 factor levels based on the 4 quartiles of the data: 1-resistant bees 2-infected below median or low infection 3-median to 75th quartile or medium infection 4- 75th quartile or high infection

Load data levels of infection

```
## The following objects are masked from map:
##
##     AvgRawSporeCount, BarcodeSequence, BeeNum, BeeSporeCount, Cage_Num,
##     Censoring, Coding.detail, Colony, Date_0, day_of_death,
##     DissectionDate, Experiment, InfectionCoding.level, LinkerPrimerSeq,
##     Notes_Spore, Notes_Surv, Sent_Quinn, SporeCounter, Treatment, Trial
```

NMDS Stress levels of infection

```
BC.m_lev <- metaMDS(OTU.m_lev, distance="bray", k=3,  trymax=1000)
```

```
## Square root transformation
## Wisconsin double standardization
## Run 0 stress 0.1786597
## Run 1 stress 0.1800688
## Run 2 stress 0.1786605
## ... Procrustes: rmse 0.0002765532  max resid 0.002251845
## ... Similar to previous best
## Run 3 stress 0.1786593
```

```
## ... New best solution
## ... Procrustes: rmse 0.0007287213  max resid 0.005973233
## ... Similar to previous best
## Run 4 stress 0.1786602
## ... Procrustes: rmse 0.0007271948  max resid 0.005371045
## ... Similar to previous best
## Run 5 stress 0.1786592
## ... New best solution
## ... Procrustes: rmse 0.0006163545  max resid 0.005126951
## ... Similar to previous best
## Run 6 stress 0.178661
## ... Procrustes: rmse 0.0003540776  max resid 0.003046744
## ... Similar to previous best
## Run 7 stress 0.1786614
## ... Procrustes: rmse 0.0008721329  max resid 0.008195728
## ... Similar to previous best
## Run 8 stress 0.1786632
## ... Procrustes: rmse 0.001063537  max resid 0.01029224
## Run 9 stress 0.1786592
## ... New best solution
## ... Procrustes: rmse 6.796113e-05  max resid 0.0004208575
## ... Similar to previous best
## Run 10 stress 0.1786593
## ... Procrustes: rmse 9.25969e-05  max resid 0.0006959062
## ... Similar to previous best
## Run 11 stress 0.1786633
## ... Procrustes: rmse 0.00129174  max resid 0.00931299
## ... Similar to previous best
## Run 12 stress 0.1786632
## ... Procrustes: rmse 0.001117427  max resid 0.009566091
## ... Similar to previous best
## Run 13 stress 0.1799826
## Run 14 stress 0.1786592
## ... New best solution
## ... Procrustes: rmse 4.106545e-05  max resid 0.0004121261
## ... Similar to previous best
## Run 15 stress 0.17866
## ... Procrustes: rmse 0.0002784296  max resid 0.002439507
## ... Similar to previous best
## Run 16 stress 0.1786597
## ... Procrustes: rmse 0.0006199267  max resid 0.006176354
## ... Similar to previous best
## Run 17 stress 0.1787823
## ... Procrustes: rmse 0.004555189  max resid 0.04446234
## Run 18 stress 0.1799832
## Run 19 stress 0.1786622
## ... Procrustes: rmse 0.001079747  max resid 0.007091498
## ... Similar to previous best
## Run 20 stress 0.1786595
## ... Procrustes: rmse 0.0005483841  max resid 0.00384774
## ... Similar to previous best
## *** Best solution repeated 5 times
```

```
stressplot(BC.m_lev)
```

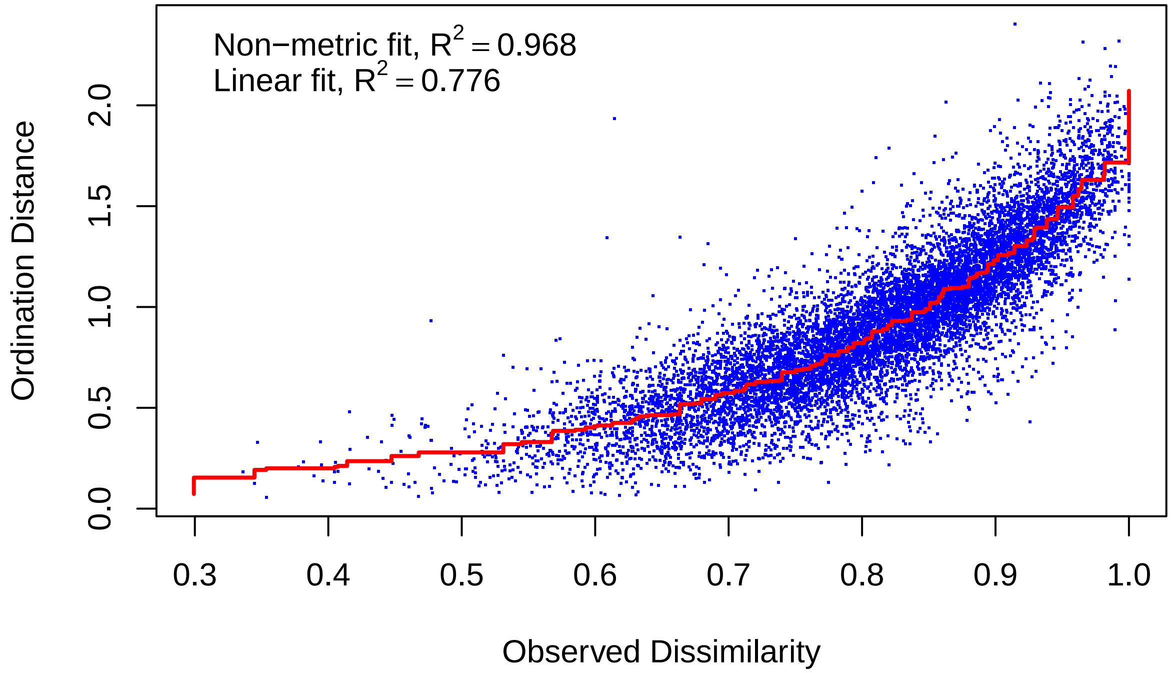


```
BC.m_lev
```

```
##
## Call:
## metaMDS(comm = OTU.m_lev, distance = "bray", k = 3, trymax = 1000)
##
## global Multidimensional Scaling using monoMDS
##
## Data:     wisconsin(sqrt(OTU.m_lev))
## Distance: bray
##
## Dimensions: 3
## Stress:     0.1786592
## Stress type 1, weak ties
## Best solution was repeated 5 times in 20 tries
## The best solution was from try 14 (random start)
## Scaling: centring, PC rotation, halfchange scaling
## Species: expanded scores based on 'wisconsin(sqrt(OTU.m_lev))'
```

NMDS Plot levels of infection

```
setEPS()
postscript("FINAL_NMDS_plot_levels.eps", horizontal = FALSE, onefile = FALSE, paper = "special", height

map_lev_of_in$infect_lev_factor<- factor(map_lev_of_in$Coding.detail, levels = c("1", "2", "3","4"))

plot(BC.m_lev, "sites",
     main ="Microbial Community Composition by Infection level")
with(map_lev_of_in,
     points(BC.m_lev,
            display = "sites",
            pch = 20,
            col = c("purple", "yellow", "orange", "red")))

legend("topleft", legend=c("resistant", "low", "medium", "high"), col=c("purple", "yellow", "orange", "


ordiellipse(
  BC.m_lev,
  map_lev_of_in$InfectionCoding.level,
  display = "sites",
  conf = 0.95,
  col = c("purple", "yellow", "orange", "red"),
  lwd = 1.5,
)

dev.off()
```

```
## pdf
##   2
```

Resistance This analysis looks at resistant versus control bees.

Load files for resistance

```
## The following objects are masked from map_lev_of_in:
##
##     AvgRawSporeCount, BarcodeSequence, BeeNum, BeeSporeCount, Cage_Num,
##     Censoring, Coding.detail, Colony, Date_0, day_of_death,
##     DissectionDate, Experiment, InfectionCoding.level, LinkerPrimerSeq,
##     Notes_Spore, Notes_Surv, Sent_Quinn, SporeCounter, Treatment, Trial
```

```
## The following objects are masked from map:
##
##     AvgRawSporeCount, BarcodeSequence, BeeNum, BeeSporeCount, Cage_Num,
##     Censoring, Coding.detail, Colony, Date_0, day_of_death,
##     DissectionDate, Experiment, InfectionCoding.level, LinkerPrimerSeq,
##     Notes_Spore, Notes_Surv, Sent_Quinn, SporeCounter, Treatment, Trial
```

NMDS Stress Resistance

```
BC.m_res <- metaMDS(OTU.m_res, distance="bray", k=3,  trymax=1000)
```

```
## Square root transformation
## Wisconsin double standardization
## Run 0 stress 0.1962248
## Run 1 stress 0.1962231
## ... New best solution
## ... Procrustes: rmse 0.0007091419  max resid 0.005133135
## ... Similar to previous best
## Run 2 stress 0.1983385
## Run 3 stress 0.1983958
## Run 4 stress 0.1962227
## ... New best solution
## ... Procrustes: rmse 0.0004328827  max resid 0.003639721
## ... Similar to previous best
## Run 5 stress 0.1999013
## Run 6 stress 0.1973818
## Run 7 stress 0.1962227
## ... Procrustes: rmse 0.0002096384  max resid 0.001524659
## ... Similar to previous best
## Run 8 stress 0.1962227
## ... Procrustes: rmse 0.0001602148  max resid 0.001120696
## ... Similar to previous best
## Run 9 stress 0.1962226
## ... New best solution
## ... Procrustes: rmse 7.681048e-05  max resid 0.0004891096
## ... Similar to previous best
## Run 10 stress 0.1962239
## ... Procrustes: rmse 0.0005882466  max resid 0.004491712
## ... Similar to previous best
## Run 11 stress 0.1982809
## Run 12 stress 0.1981252
## Run 13 stress 0.1962229
## ... Procrustes: rmse 0.0002369332  max resid 0.001922362
## ... Similar to previous best
## Run 14 stress 0.2003596
## Run 15 stress 0.1973827
## Run 16 stress 0.2003622
## Run 17 stress 0.1973773
## Run 18 stress 0.1993441
## Run 19 stress 0.1962239
## ... Procrustes: rmse 0.0007595528  max resid 0.006570268
## ... Similar to previous best
## Run 20 stress 0.1981322
## *** Best solution repeated 4 times
```

```
stressplot(BC.m_res)
```

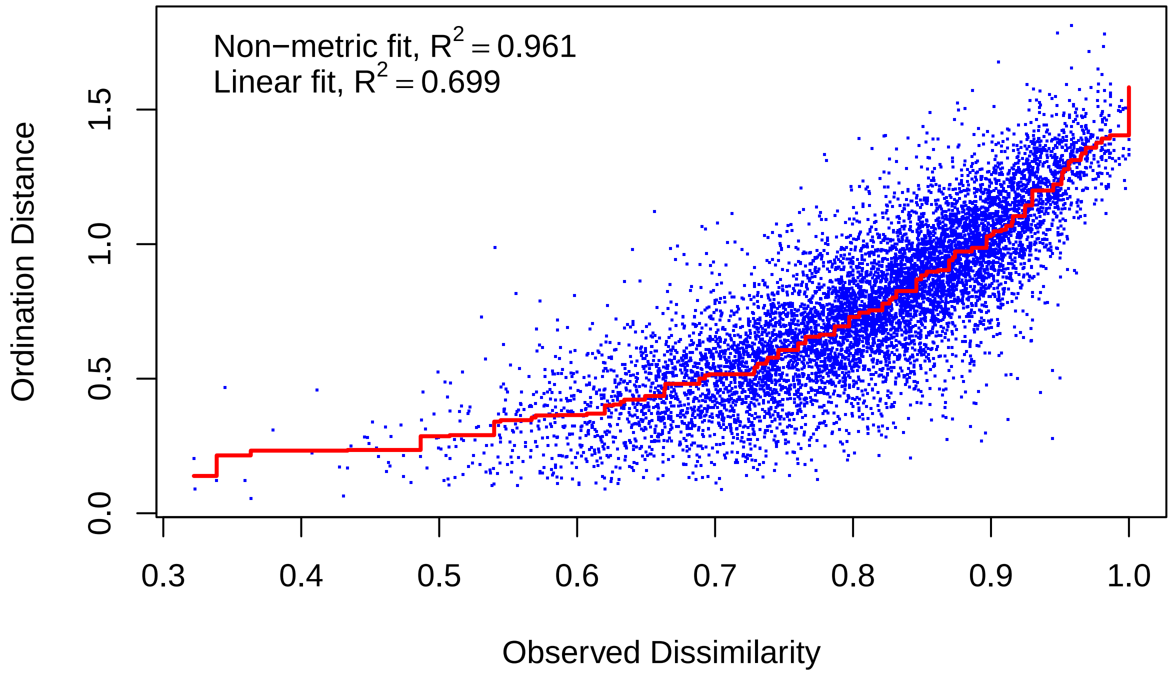


```
BC.m_res
```

```
##
## Call:
## metaMDS(comm = OTU.m_res, distance = "bray", k = 3, trymax = 1000)
##
## global Multidimensional Scaling using monoMDS
##
## Data:     wisconsin(sqrt(OTU.m_res))
## Distance: bray
##
## Dimensions: 3
## Stress:     0.1962226
## Stress type 1, weak ties
## Best solution was repeated 4 times in 20 tries
## The best solution was from try 9 (random start)
## Scaling: centring, PC rotation, halfchange scaling
## Species: expanded scores based on 'wisconsin(sqrt(OTU.m_res))'
```

NMDS Plot Resistance

```
setEPS()
postscript("FINAL_NMDS_plot_resistvcont.eps", horizontal = FALSE, onefile = FALSE, paper = "special", he

plot(BC.m_res, display = "sites")
```

```
orditorp(BC.m_res, "sites")

map_resist$infect_lev_factor<- factor(map_resist$Coding.detail, levels = c("0", "1"))

plot(BC.m_res, "sites",
     main ="Microbial Community Composition by Control vs Resistant")
with(map_resist,
     points(BC.m_res,
            display = "sites",
            pch = 20,
            col = c("black", "purple")))

legend("bottomright", legend=c("Control","Resistant"), col=c("black", "purple"), pch=20, cex = 0.75)


ordiellipse(
  BC.m_res,
  map_resist$InfectionCoding.level,
  display = "sites",
  conf = 0.95,
  col = c("black", "purple"),
  lwd = 2.5,
)

dev.off()
```

```
## pdf
##   2
```

Adonis and Beta Dispersion Statistics

Treatment Versus Control I first calculate bray curtis distance matrices, then run Adonis

Adonis treatment versus control

```
set.seed(22)
BC.dist <- vegdist(OTU.m, distance="bray")
perms <- with(map, how(nperm = 1000, blocks = Colony))
adonis2(formula = BC.dist ~ Treatment, permutations = perms, data = map, pairwise = TRUE)
```

```
## Permutation test for adonis under reduced model
## Terms added sequentially (first to last)
## Blocks:  Colony
## Permutation: free
## Number of permutations: 1000
##
## adonis2(formula = BC.dist ~ Treatment, data = map, permutations = perms, pairwise = TRUE)
##            Df SumOfSqs      R2      F   Pr(>F)
## Treatment   1    1.208 0.01907 4.4335 0.000999 ***
## Residual  228   62.134 0.98093
## Total     229   63.342 1.00000
## ---
## Signif. codes:  0 '***' 0.001 '**' 0.01 '*' 0.05 '.' 0.1 ' ' 1
```

Beta diversity is significantly different between treat and control (df=1,F=4.4335,P=0.000999 ***)

Beta Dispersion Treatment versus Control

```
set.seed(22)
disp = betadisper(BC.dist, map$Treatment)
perms.bd <- with(map, how(nperm = 1000, blocks = Colony))
p.w.bd <- permutest(disp, pairwise=TRUE, permutations=perms.bd)
p.w.bd
```

```
##
## Permutation test for homogeneity of multivariate dispersions
## Blocks:  Colony
## Permutation: free
## Number of permutations: 1000
##
## Response: Distances
##            Df  Sum Sq  Mean Sq      F N.Perm Pr(>F)
## Groups      1 0.07732 0.077316 6.2705   1000 0.1119
## Residuals 228 2.81125 0.012330
##
## Pairwise comparisons:
## (Observed p-value below diagonal, permuted p-value above diagonal)
##          Cont    Exp
## Cont           0.3646
## Exp  0.012976
```

Beta dispersion is not significantly different between treat and control.

Levels of Infection Adonis Levels of Infection

```
set.seed(22)
BC.dist_lev_in <- vegdist(OTU.m_lev, distance="bray")
perms_lev_in <- with(map_lev_of_in, how(nperm = 1000, blocks = Colony))
adonis2(formula = BC.dist_lev_in ~ Coding.detail, permutations = perms_lev_in, data = map_lev_of_in, pa
```

```
## Permutation test for adonis under reduced model
## Terms added sequentially (first to last)
## Blocks:  Colony
## Permutation: free
## Number of permutations: 1000
##
## adonis2(formula = BC.dist_lev_in ~ Coding.detail, data = map_lev_of_in, permutations = perms_lev_in,
##                Df SumOfSqs      R2      F  Pr(>F)
## Coding.detail   3    1.669 0.04029 1.9873 0.03297 *
## Residual      142   39.755 0.95971
## Total         145   41.424 1.00000
## ---
## Signif. codes:  0 '***' 0.001 '**' 0.01 '*' 0.05 '.' 0.1 ' ' 1
```

```
map_lev_of_in$Coding.detail=as.factor(map_lev_of_in$Coding.detail)
adonis.pair(BC.dist_lev_in, map_lev_of_in$Coding.detail, nper = 1000, corr.method = "fdr")
```

```
## 'adonis' will be deprecated: use 'adonis2' instead
## 'adonis' will be deprecated: use 'adonis2' instead
## 'adonis' will be deprecated: use 'adonis2' instead
## 'adonis' will be deprecated: use 'adonis2' instead
## 'adonis' will be deprecated: use 'adonis2' instead
## 'adonis' will be deprecated: use 'adonis2' instead
```

```
##                                               combination SumsOfSqs   MeanSqs
## 1                  75th quartile <-> infected below median 0.5528462 0.5528462
## 2                75th quartile <-> median to 75th quartile 0.2222620 0.2222620
## 3          75th quartile <-> Resistant bees (no infection) 0.6266268 0.6266268
## 4        infected below median <-> median to 75th quartile 0.6301716 0.6301716
## 5   infected below median <-> Resistant bees (no infection) 0.2594787 0.2594787
## 6 median to 75th quartile <-> Resistant bees (no infection) 0.9872517 0.9872517
##     F.Model         R2     P.value P.value.corrected
## 1 1.9973359 0.03170508 0.029970030       0.044955045
## 2 0.8817955 0.01244037 0.575424575       0.604395604
## 3 2.1197013 0.02613054 0.021978022       0.043956044
## 4 2.4206541 0.03700137 0.007992008       0.023976024
## 5 0.8449426 0.01159919 0.604395604       0.604395604
## 6 3.4965197 0.04138064 0.000999001       0.005994006
```

Adonis shows significant differences in beta diversity for differing levels of infection (df=3, F=1.9873, P=0.03297 *). The specific pairwise comparisons are all significant except for infected below median <-> Resistant bees (no infection) and 75th quartile <-> median to 75th quartile.

Beta Dispersion Levels of Infection

```
set.seed(22)
disp_lev = betadisper(BC.dist_lev_in, map_lev_of_in$Coding.detail)
perms.bd_lev <- with(map_lev_of_in, how(nperm = 1000, blocks = Colony))
p.w.bd_lev <- permutest(disp_lev, pairwise=TRUE, permutations=perms.bd_lev)
p.w.bd_lev
```

```
##
## Permutation test for homogeneity of multivariate dispersions
## Blocks:  Colony
## Permutation: free
## Number of permutations: 1000
##
## Response: Distances
##            Df  Sum Sq  Mean Sq      F N.Perm  Pr(>F)
## Groups      3 0.13176 0.043922 3.6076   1000 0.04096 *
## Residuals 142 1.72879 0.012175
## ---
## Signif. codes:  0 '***' 0.001 '**' 0.01 '*' 0.05 '.' 0.1 ' ' 1
##
## Pairwise comparisons:
## (Observed p-value below diagonal, permuted p-value above diagonal)
##                                 75th quartile infected below median
##  75th quartile                                            0.4035964
## infected below median               0.4182412
## median to 75th quartile             0.3190399             0.0677096
## Resistant bees (no infection)       0.0500867             0.2889790
```

```
##                                median to 75th quartile
##  75th quartile                              0.2677323
## infected below median                       0.0269730
## median to 75th quartile
## Resistant bees (no infection)               0.0020857
##                                Resistant bees (no infection)
##  75th quartile                                       0.1279
## infected below median                                0.7812
## median to 75th quartile                              0.0170
## Resistant bees (no infection)
```

Beta dispersion significant for differing levels of infection (df=3,F=3.6076, P=0.04096 *). In the manuscript we use the observed p-values for the pairwise comparisons (below the diagonal)

Resistance Adonis Resistance

```
set.seed(22)
BC.dist_res <- vegdist(OTU.m_res, distance="bray")
perms_res <- with(map_resist, how(nperm = 1000, blocks = Colony))
adonis2(formula = BC.dist_res ~ Treatment, permutations = perms_res, data = map_resist, pairwise = TRUE
```

```
## Permutation test for adonis under reduced model
## Terms added sequentially (first to last)
## Blocks:  Colony
## Permutation: free
## Number of permutations: 1000
##
## adonis2(formula = BC.dist_res ~ Treatment, data = map_resist, permutations = perms_res, pairwise = TR
##            Df SumOfSqs      R2      F   Pr(>F)
## Treatment   1    0.809 0.02285 2.9467 0.000999 ***
## Residual  126   34.605 0.97715
## Total     127   35.414 1.00000
## ---
## Signif. codes:  0 '***' 0.001 '**' 0.01 '*' 0.05 '.' 0.1 ' ' 1
```

Beta diversity significantly different between resistance and control bees (df=1,F=2.9467,P=0.000999 **)

Beta Dispersion Resistance

```
set.seed(22)
disp_res = betadisper(BC.dist_res, map_resist$Coding.detail)
perms.bd_res <- with(map_resist, how(nperm = 1000, blocks = Colony))
p.w.bd_res <- permutest(disp_res, pairwise=TRUE, permutations=perms.bd_res)
p.w.bd_res
```

```
##
## Permutation test for homogeneity of multivariate dispersions
## Blocks:  Colony
## Permutation: free
## Number of permutations: 1000
##
## Response: Distances
```

```
##            Df Sum Sq  Mean Sq     F N.Perm   Pr(>F)
## Groups      1 0.1480 0.147997 13.58   1000 0.000999 ***
## Residuals 126 1.3732 0.010898
## ---
## Signif. codes:  0 '***' 0.001 '**' 0.01 '*' 0.05 '.' 0.1 ' ' 1
##
## Pairwise comparisons:
## (Observed p-value below diagonal, permuted p-value above diagonal)
##                                 Resistant bees (no infection) uninfected control
## Resistant bees (no infection)                                           0.0609
## uninfected control                                 0.00033818
```

Beta dispersion significant differences between control and resistant bees (df=1,F=13.58,P=0.000999 ***).

Alpha Diversity

Treatment Versus Control Load files Treatment Versus Control

I load in observed_otu alpha diversity files generated in qiime2 with raw ASVs. They went through dada2 in qiime2 so they are ASVs not OTUs.

```
K_W <- read.table("alpha-diversity_pruned.txt", header = T, fill=TRUE, row.names = 1, sep='\t')
AlDiv.df <- as.data.frame(K_W)
```

Data Spread Treatment Versus Control

Now I'm going to check whether the ASVs are normally distributed.

```
hist(AlDiv.df$observed_otus)
```

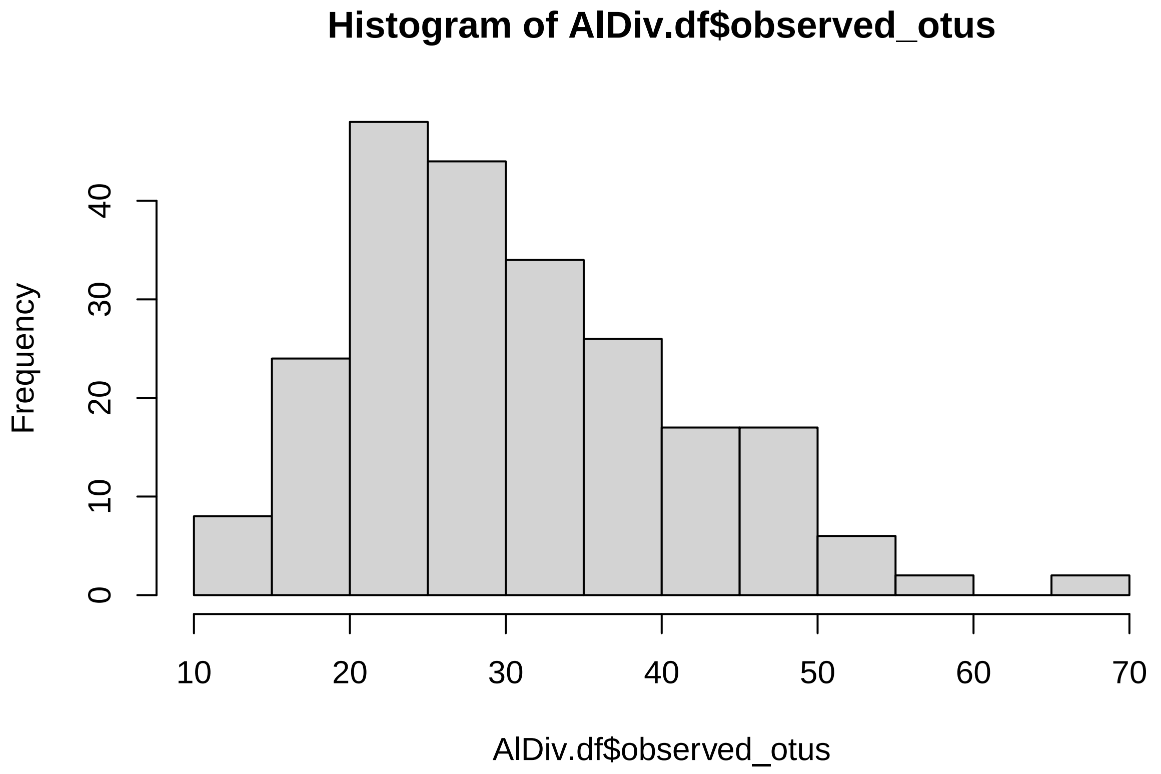

They have a moderate positive skew so I'm going to sqrt transform. I checked the model assumptions without transforming and the residuals were not homoscedastic.

```
AlDiv.df.sqtran <- sqrt(AlDiv.df$observed_otus)
hist(AlDiv.df.sqtran)
```

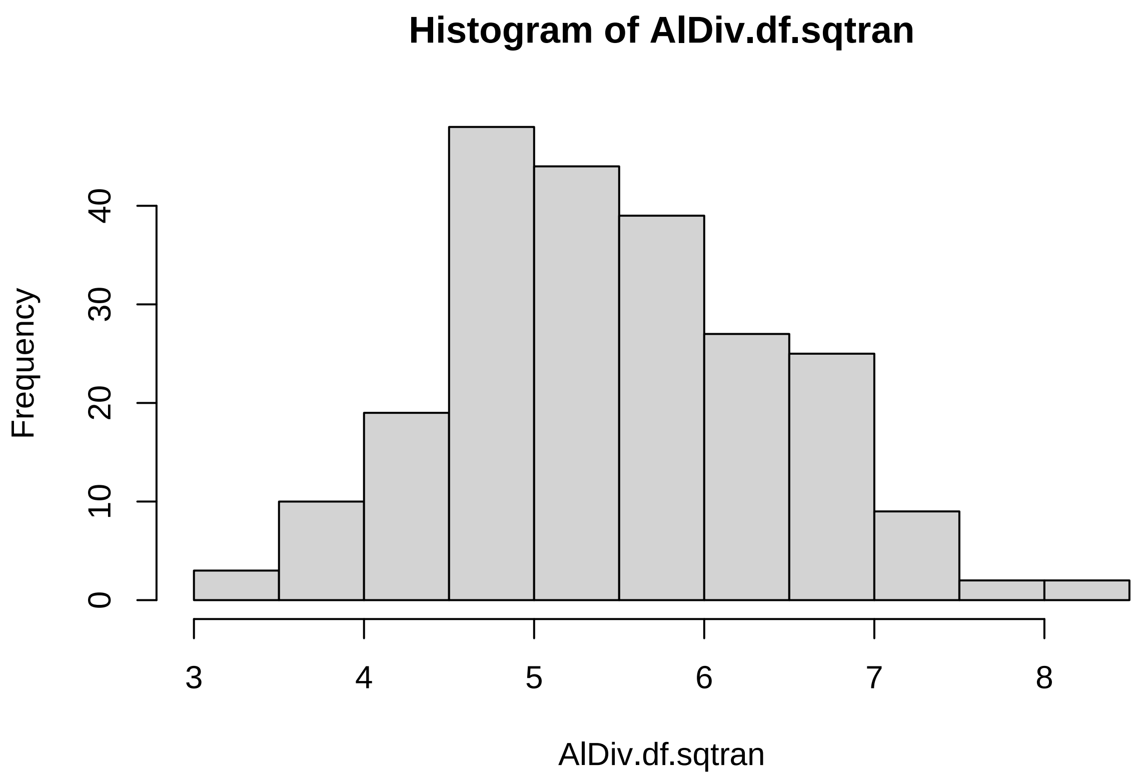

LMM model Treatment Versus Control

```
m1.0<-lmer(AlDiv.df.sqtran ~ Treatment + (1|Colony) + (1|Cage_Num), data=AlDiv.df, na.action="na.omit",
summary(m1.0)
```

```
## Linear mixed model fit by maximum likelihood  ['lmerMod']
## Formula: AlDiv.df.sqtran ~ Treatment + (1 | Colony) + (1 | Cage_Num)
##    Data: AlDiv.df
##
##      AIC      BIC   logLik deviance df.resid
##    403.0    418.8   -196.5    393.0      171
##
## Scaled residuals:
##     Min      1Q  Median      3Q     Max
## -2.6294 -0.6341  0.0084  0.6156  3.2352
##
## Random effects:
##  Groups   Name        Variance Std.Dev.
##  Cage_Num (Intercept) 0.1122   0.3349
##  Colony   (Intercept) 0.2173   0.4662
##  Residual             0.4586   0.6772
## Number of obs: 176, groups:  Cage_Num, 15; Colony, 9
##
## Fixed effects:
##             Estimate Std. Error t value
## (Intercept)   5.2469     0.2497   21.01
```

```
## TreatmentExp   0.4603     0.2064    2.23
##
## Correlation of Fixed Effects:
##              (Intr)
## TreatmntExp -0.594
```

```
Anova(m1.0)
```

```
## Analysis of Deviance Table (Type II Wald chisquare tests)
##
## Response: AlDiv.df.sqtran
##            Chisq Df Pr(>Chisq)
## Treatment 4.9726  1    0.02575 *
## ---
## Signif. codes:  0 '***' 0.001 '**' 0.01 '*' 0.05 '.' 0.1 ' ' 1
```

Alpha diversity significantly affected by the treatment (P=0.02575 *).

Test Assumptions for LMM model Treatment Versus Control

```
plot(m1.0)
```

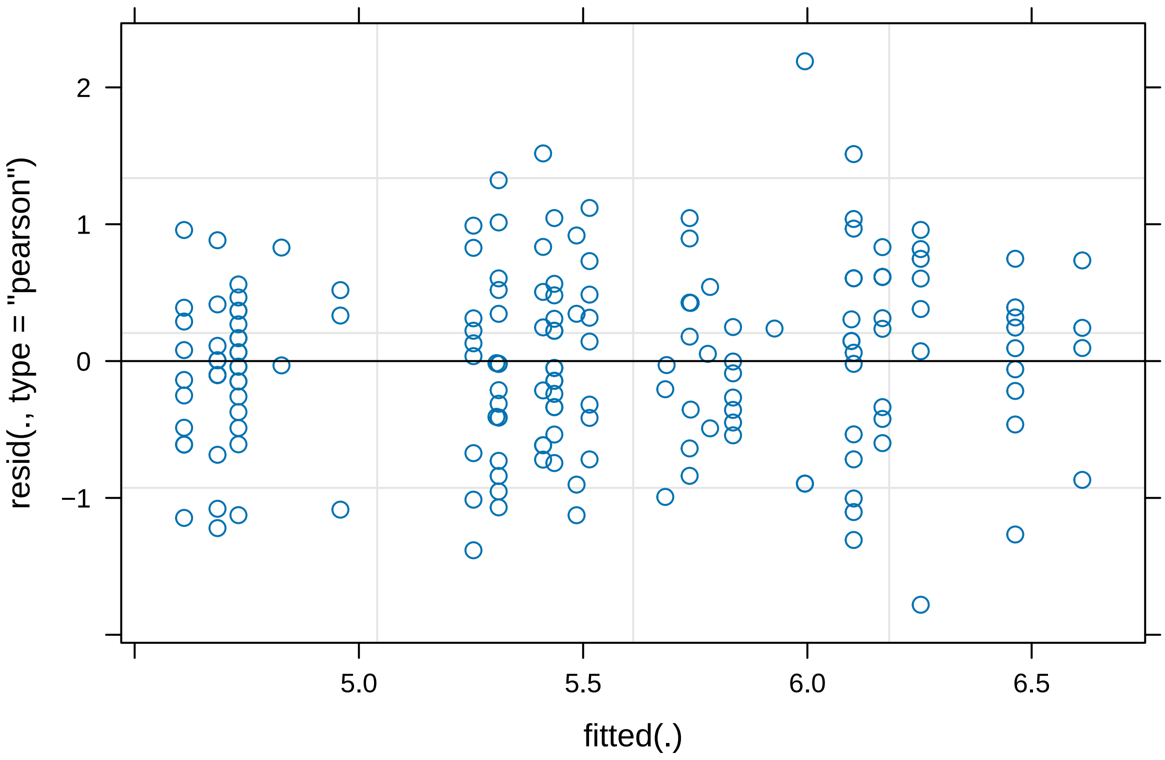


```
summary(m1.0)
```

```
## Linear mixed model fit by maximum likelihood  ['lmerMod']
## Formula: AlDiv.df.sqtran ~ Treatment + (1 | Colony) + (1 | Cage_Num)
##    Data: AlDiv.df
##
##      AIC      BIC   logLik deviance df.resid
##    403.0    418.8   -196.5    393.0      171
##
## Scaled residuals:
##     Min      1Q  Median      3Q     Max
## -2.6294 -0.6341  0.0084  0.6156  3.2352
##
## Random effects:
##  Groups   Name        Variance Std.Dev.
##  Cage_Num (Intercept) 0.1122   0.3349
##  Colony   (Intercept) 0.2173   0.4662
##  Residual             0.4586   0.6772
## Number of obs: 176, groups:  Cage_Num, 15; Colony, 9
##
## Fixed effects:
##              Estimate Std. Error t value
## (Intercept)    5.2469     0.2497   21.01
## TreatmentExp   0.4603     0.2064    2.23
##
## Correlation of Fixed Effects:
##             (Intr)
## TreatmntExp -0.594
```

```
qqnorm(resid(m1.0))
```

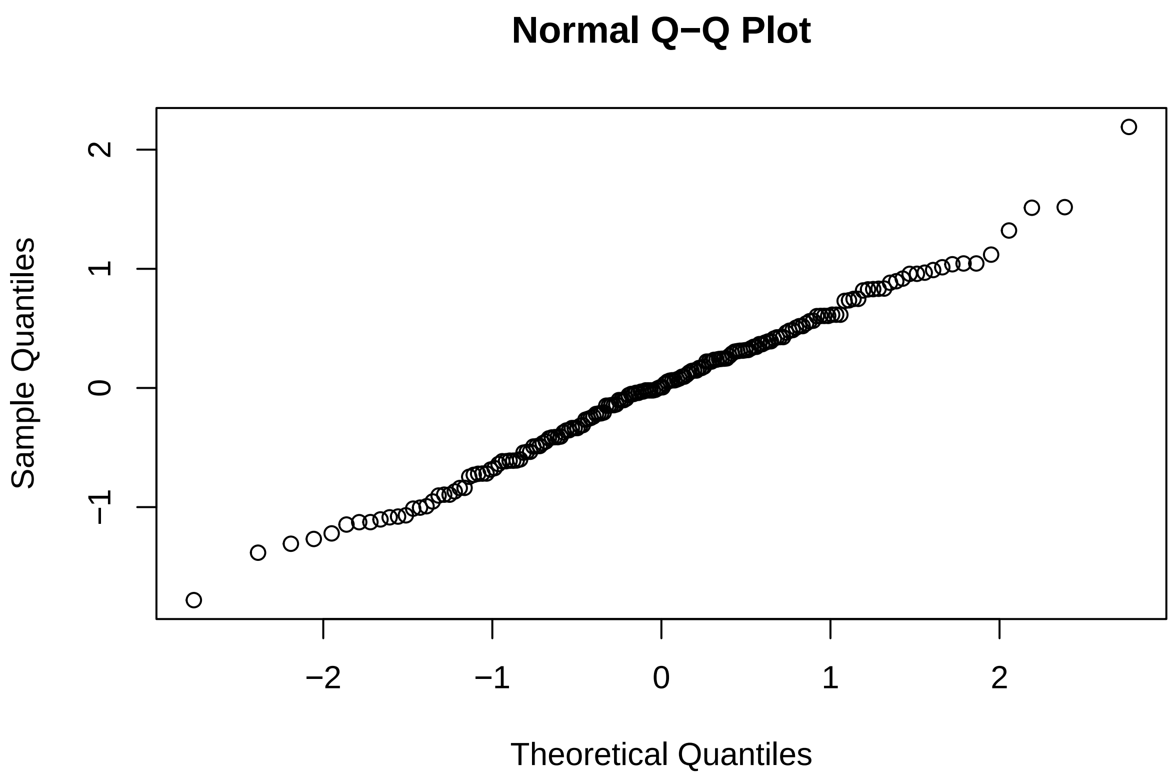


```
simoutbin500.1<-simulateResiduals(fittedModel=m1.0, n=250)
plot(simoutbin500.1)
```

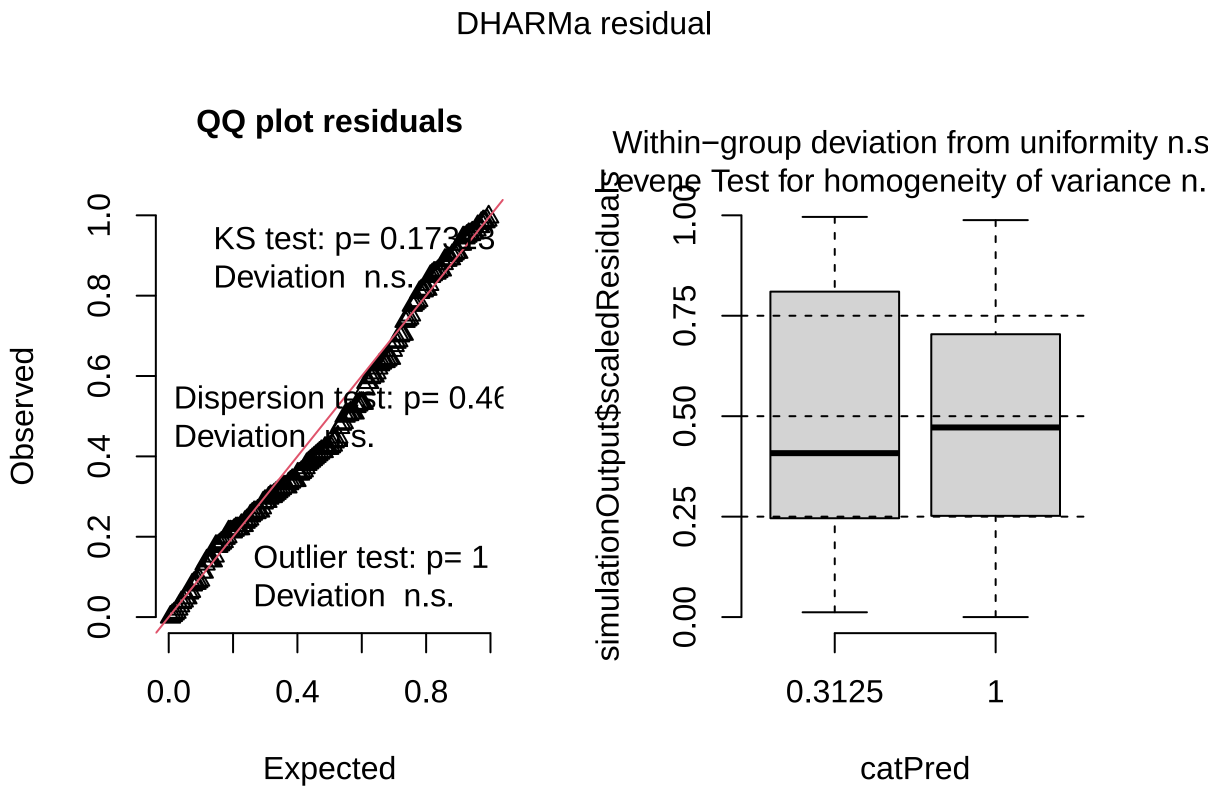

No assumptions were violated in our model!

Resistance Load files Resistance

```
K_W_res <- read.table("Alpha_div_resist.txt", header = T, fill=TRUE, row.names = 1, sep='\t')
AlDiv.df_res <- as.data.frame(K_W_res)
```

Data Spread Resistance

Now I'm going to check whether the ASVs are normally distributed.

```
hist(AlDiv.df_res$observed_otus)
```

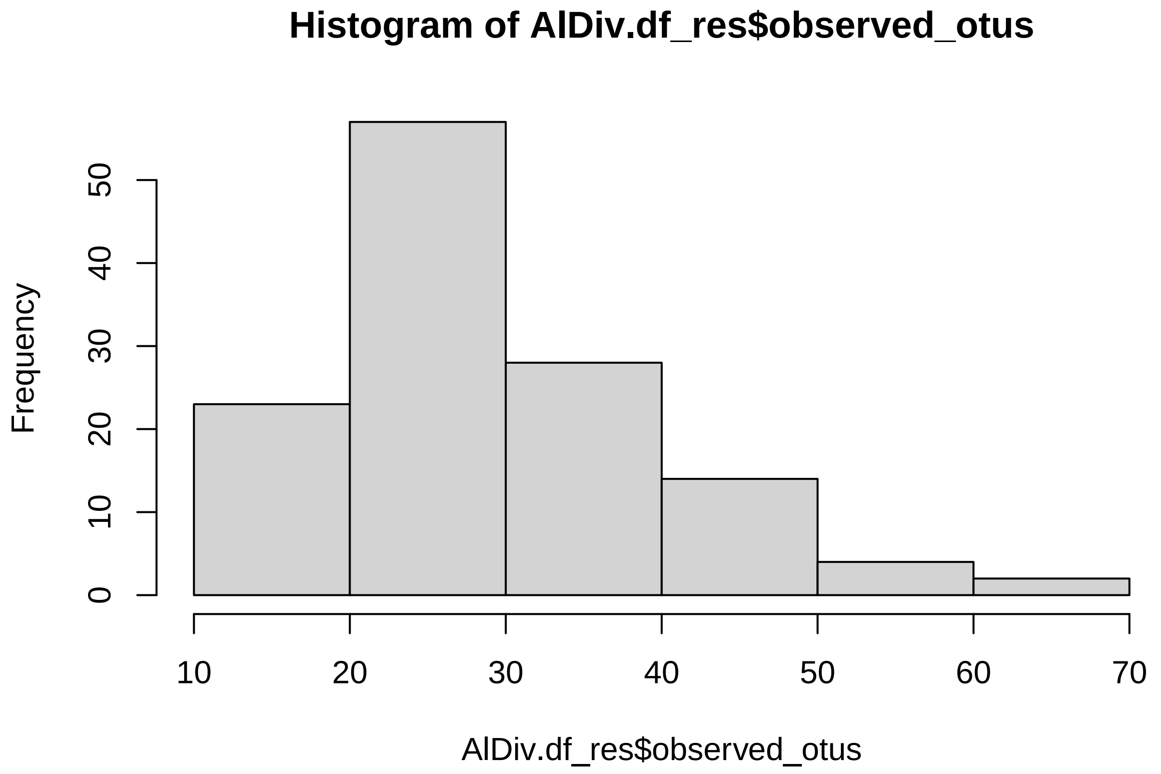

There was a slight positive skew so I square-root transformed the data.

```
AlDiv.df.sqtran_res <- sqrt(AlDiv.df_res$observed_otus)
hist(AlDiv.df.sqtran_res)
```

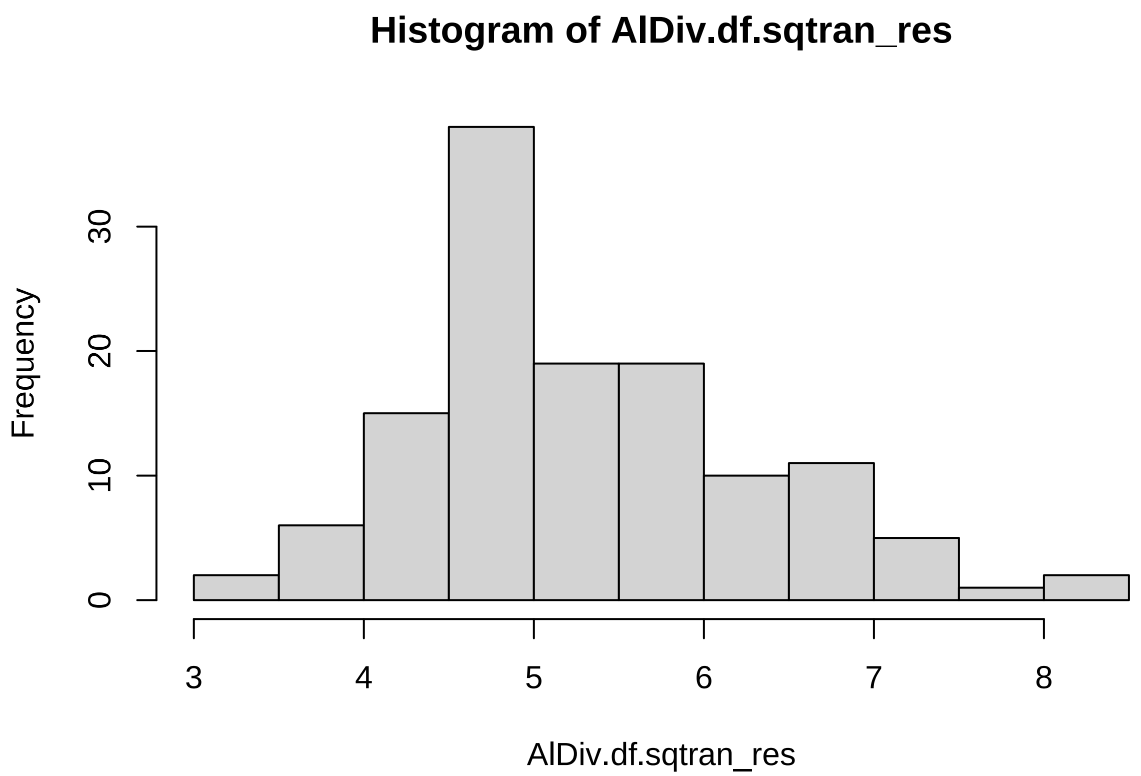

LMM model Resistance

```
m2.0<-lmer(AlDiv.df.sqtran_res ~ Code + (1|Colony) + (1|Cage_Num), data=AlDiv.df_res, na.action="na.omi
summary(m2.0)
```

```
## Linear mixed model fit by maximum likelihood  ['lmerMod']
## Formula: AlDiv.df.sqtran_res ~ Code + (1 | Colony) + (1 | Cage_Num)
##    Data: AlDiv.df_res
##
##      AIC      BIC   logLik deviance df.resid
##    243.3    256.3   -116.7    233.3       95
##
## Scaled residuals:
##      Min       1Q   Median       3Q      Max
## -2.58113 -0.58366  0.04439  0.62715  2.88327
##
## Random effects:
##  Groups   Name        Variance Std.Dev.
##  Cage_Num (Intercept) 0.05999  0.2449
##  Colony   (Intercept) 0.24807  0.4981
##  Residual             0.50559  0.7111
## Number of obs: 100, groups:  Cage_Num, 11; Colony, 8
##
## Fixed effects:
##             Estimate Std. Error t value
## (Intercept)  5.45332    0.25796   21.14
```

```
## Code         0.06405    0.29130    0.22
##
## Correlation of Fixed Effects:
##      (Intr)
## Code -0.531
```

```r
Anova(m2.0)
```

```
## Analysis of Deviance Table (Type II Wald chisquare tests)
##
## Response: AlDiv.df.sqtran_res
##       Chisq Df Pr(>Chisq)
## Code 0.0484  1      0.826
```

Not significant, but need to check model assumptions.

```r
plot(m2.0)
```

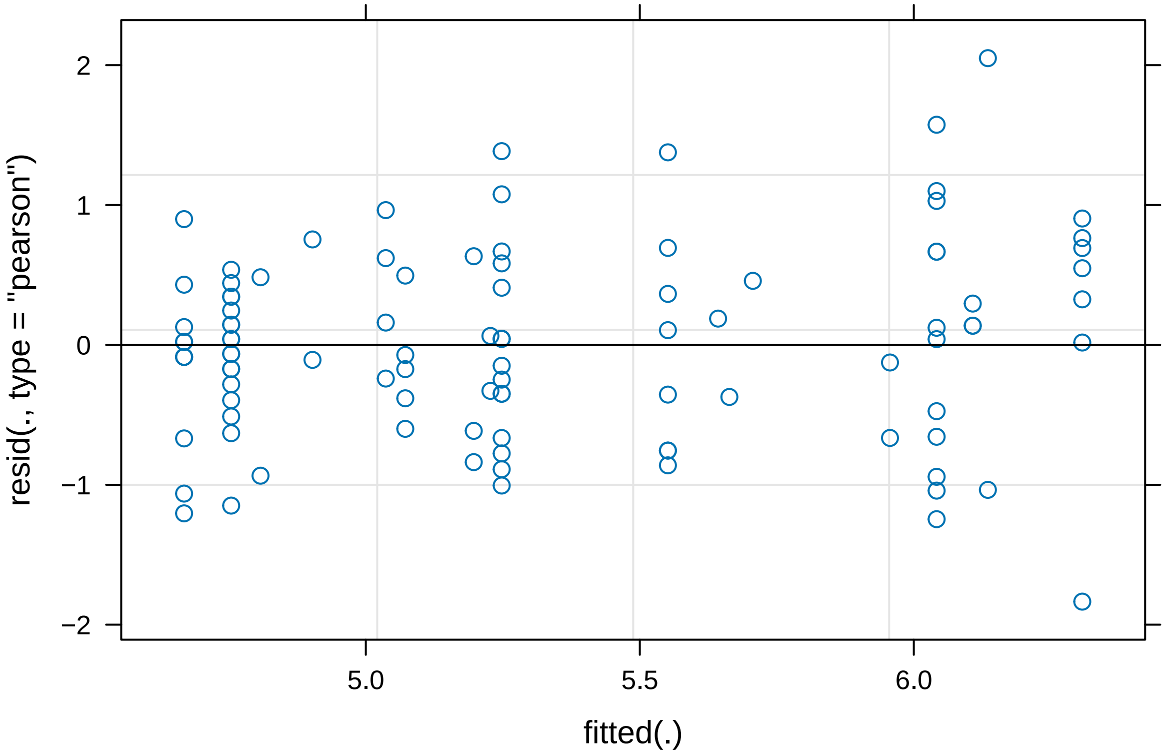


```r
summary(m2.0)
```

```
## Linear mixed model fit by maximum likelihood  ['lmerMod']
## Formula: AlDiv.df.sqtran_res ~ Code + (1 | Colony) + (1 | Cage_Num)
##    Data: AlDiv.df_res
##
```

```
##      AIC      BIC   logLik deviance df.resid
##    243.3    256.3   -116.7    233.3       95
##
## Scaled residuals:
##      Min       1Q   Median       3Q      Max
## -2.58113 -0.58366  0.04439  0.62715  2.88327
##
## Random effects:
##  Groups   Name        Variance Std.Dev.
##  Cage_Num (Intercept) 0.05999  0.2449
##  Colony   (Intercept) 0.24807  0.4981
##  Residual             0.50559  0.7111
## Number of obs: 100, groups:  Cage_Num, 11; Colony, 8
##
## Fixed effects:
##             Estimate Std. Error t value
## (Intercept)  5.45332    0.25796   21.14
## Code         0.06405    0.29130    0.22
##
## Correlation of Fixed Effects:
##      (Intr)
## Code -0.531
```

```
qqnorm(resid(m2.0))
```

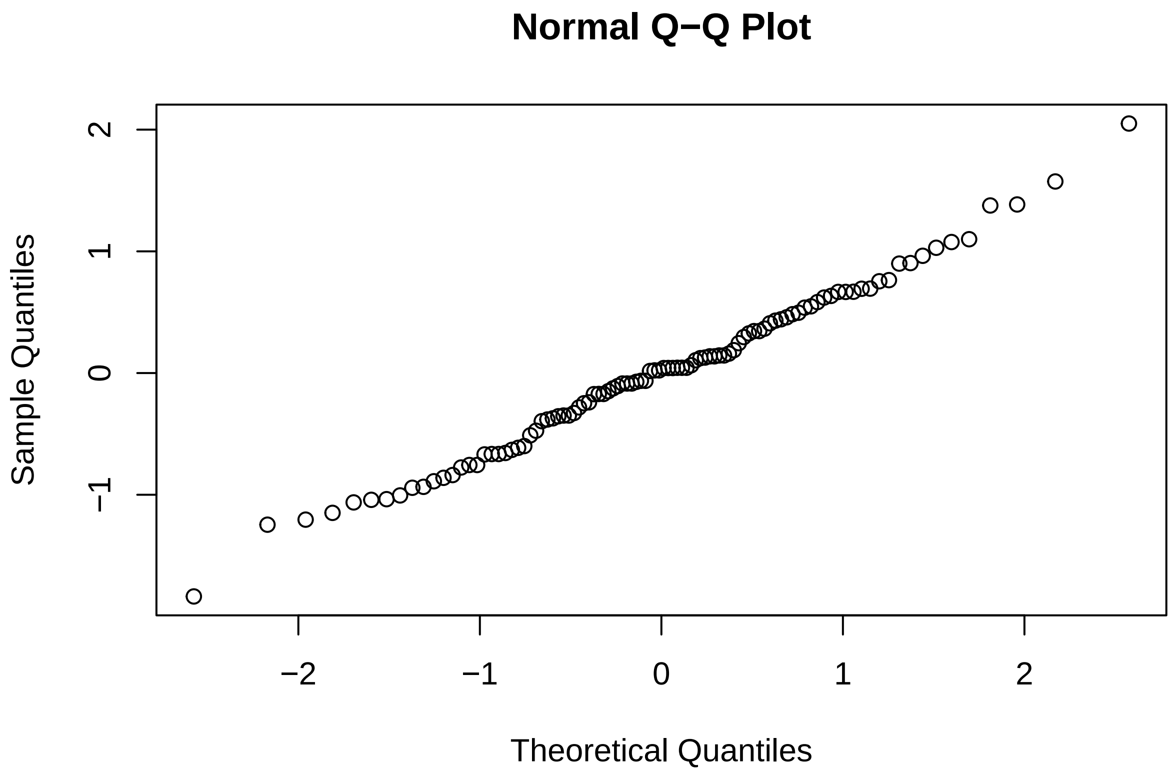

```
simoutbin500.2<-simulateResiduals(fittedModel=m2.0, n=250)
plot(simoutbin500.2)
```

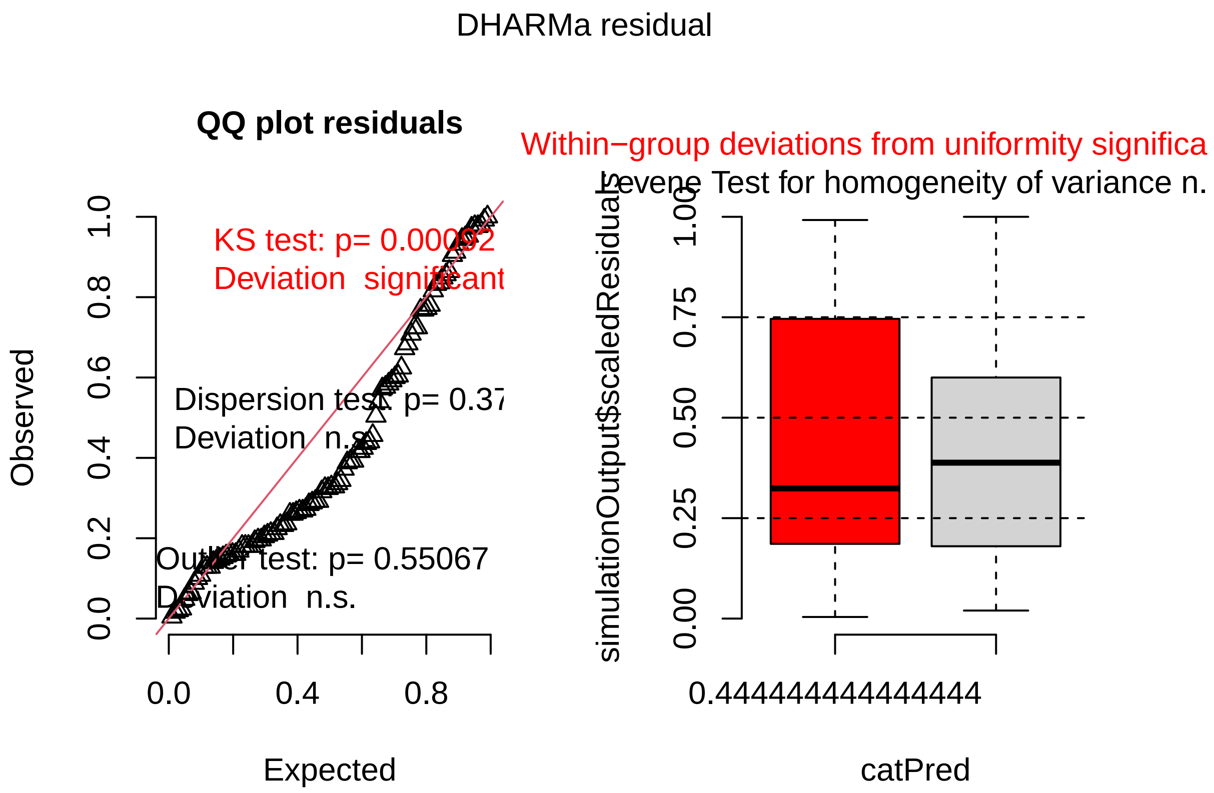

Model violated several assumptions so I just did a regular linear model which resolved those.

```
m2.1<-lm(AlDiv.df.sqtran_res ~ Code, data=AlDiv.df_res, na.action="na.omit")
summary(m2.1)
```

```
##
## Call:
## lm(formula = AlDiv.df.sqtran_res ~ Code, data = AlDiv.df_res,
##     na.action = "na.omit")
##
## Residuals:
##     Min      1Q  Median      3Q     Max
## -1.9235 -0.6972 -0.2394  0.5916  2.9790
##
## Coefficients:
##             Estimate Std. Error t value Pr(>|t|)
## (Intercept)   5.3876     0.1078  49.979   <2e-16 ***
## Code         -0.1482     0.1818  -0.815    0.416
## ---
## Signif. codes:  0 '***' 0.001 '**' 0.01 '*' 0.05 '.' 0.1 ' ' 1
##
## Residual standard error: 0.9821 on 126 degrees of freedom
```

```
## Multiple R-squared:  0.005247,   Adjusted R-squared:  -0.002648
## F-statistic: 0.6646 on 1 and 126 DF,  p-value: 0.4165
```

No significant effect.

```
plot(m2.1)
```

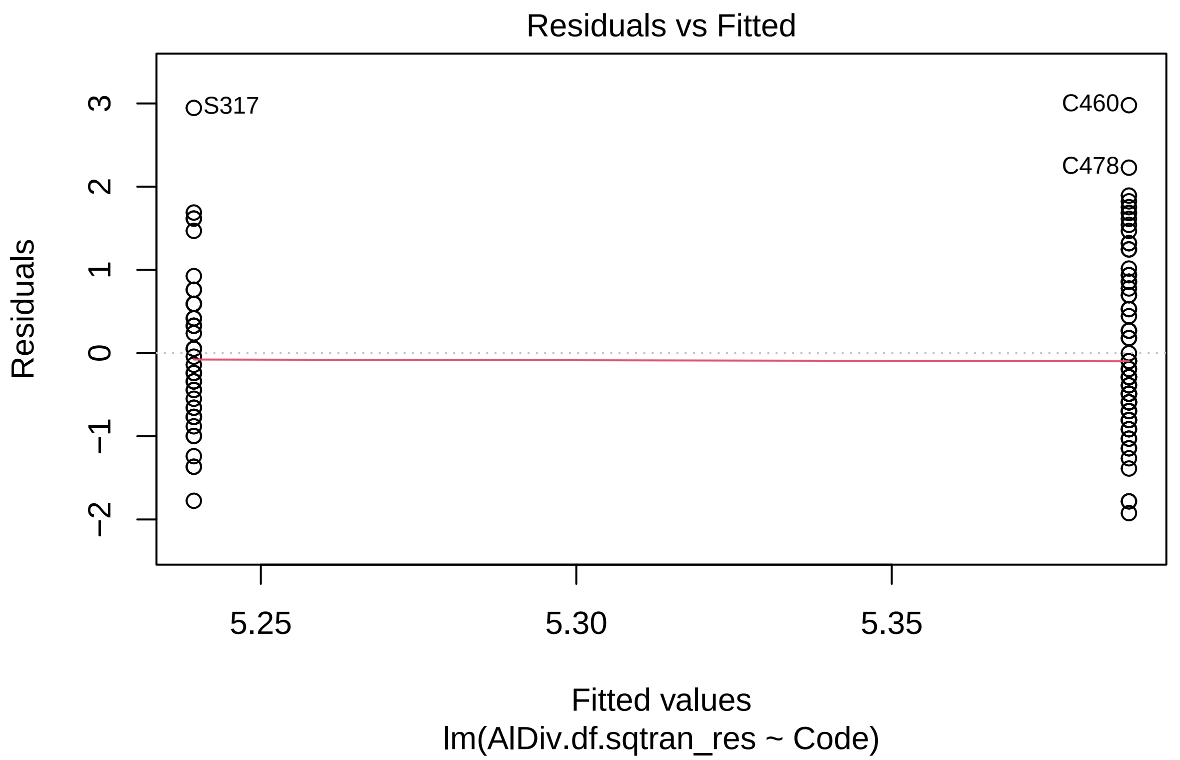

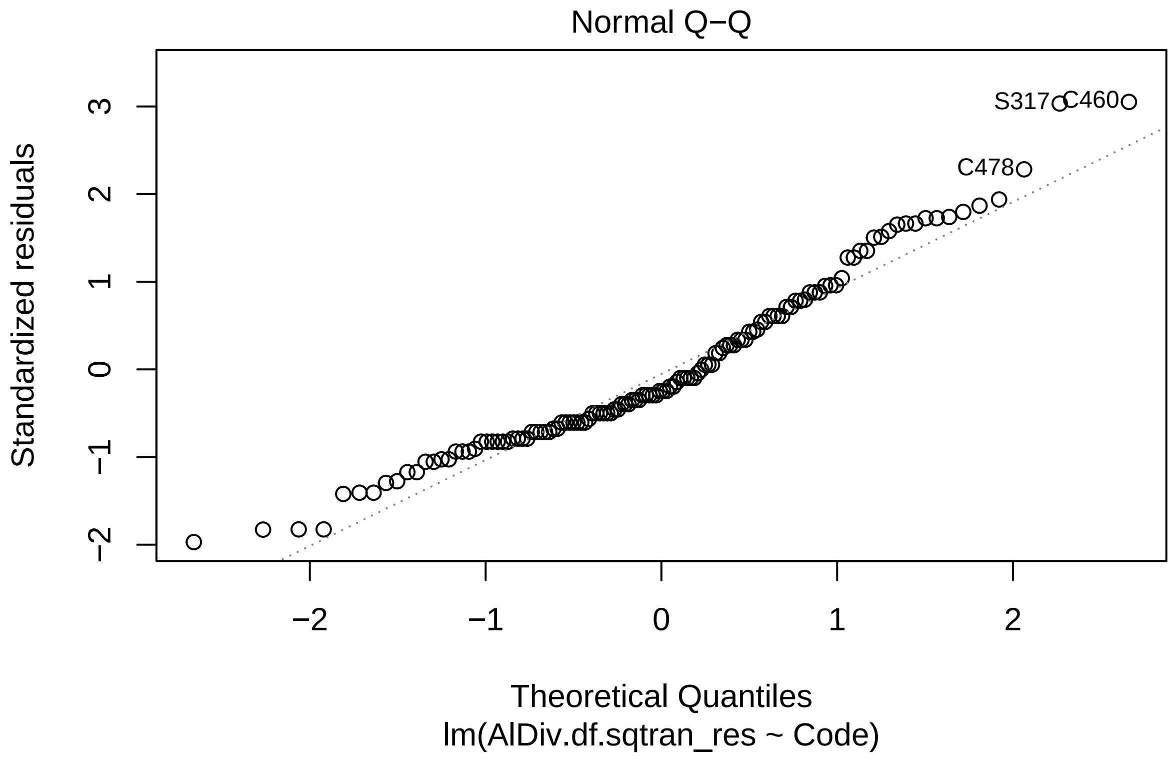
Normal Q−Q
Standardized residuals
Theoretical Quantiles
lm(AlDiv.df.sqtran_res ~ Code)
S317
C460
C478
3
2
1
0
−1
−2
−2
−1
0
1
2

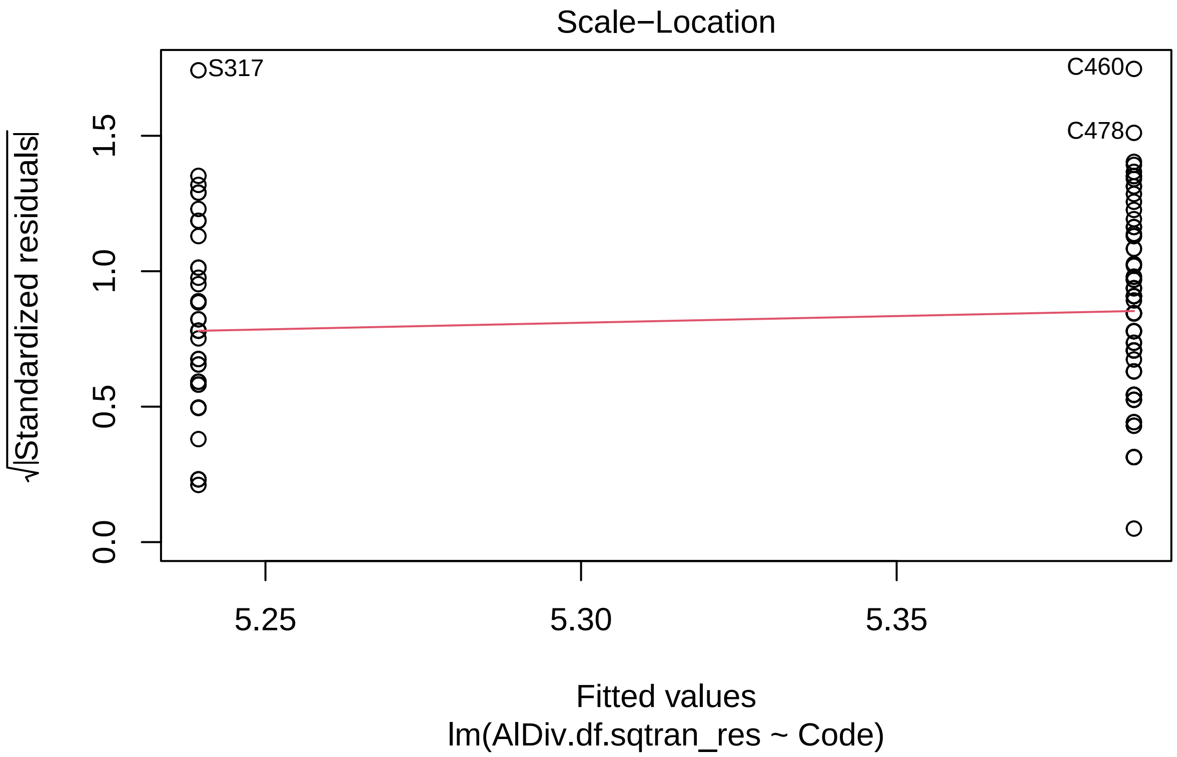
Scale−Location
√|Standardized residuals|
0.0
0.5
1.0
1.5
S317
C460
C478
5.25
5.30
5.35
Fitted values
lm(AlDiv.df.sqtran_res ~ Code)

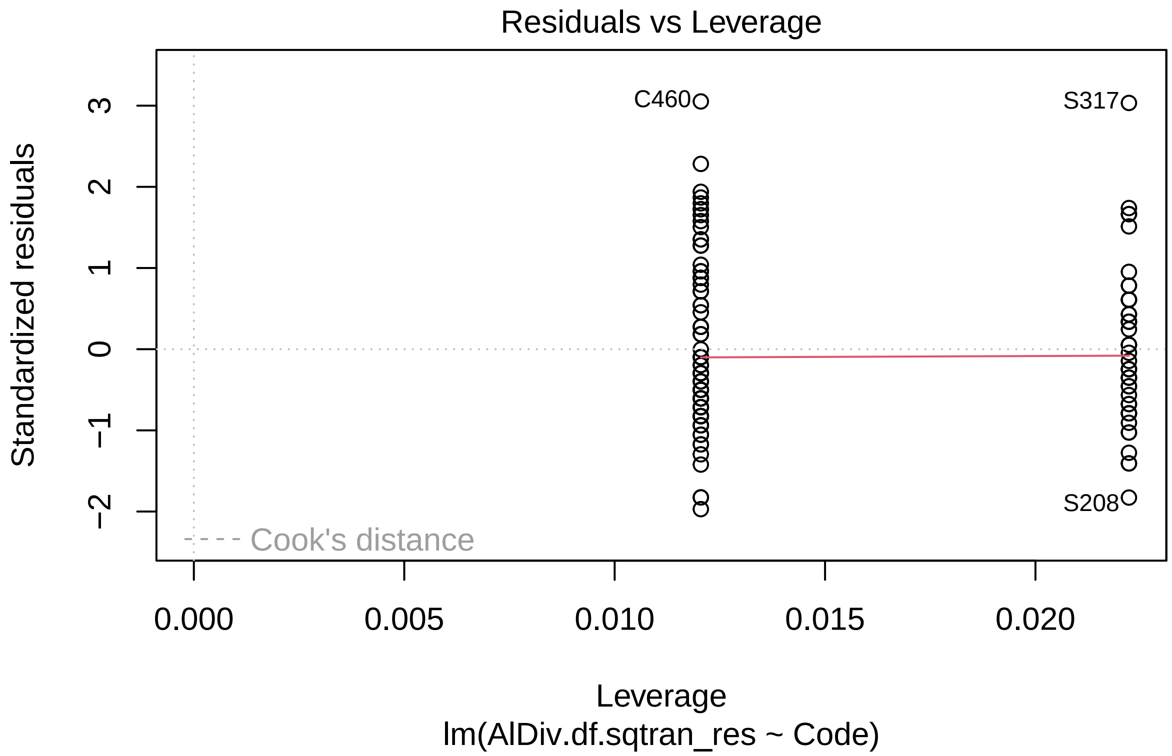


```
summary(m2.1)
```

```
##
## Call:
## lm(formula = AlDiv.df.sqtran_res ~ Code, data = AlDiv.df_res,
##     na.action = "na.omit")
##
## Residuals:
##     Min      1Q  Median      3Q     Max
## -1.9235 -0.6972 -0.2394  0.5916  2.9790
##
## Coefficients:
##             Estimate Std. Error t value Pr(>|t|)
## (Intercept)   5.3876     0.1078  49.979   <2e-16 ***
## Code         -0.1482     0.1818  -0.815    0.416
## ---
## Signif. codes:  0 '***' 0.001 '**' 0.01 '*' 0.05 '.' 0.1 ' ' 1
##
## Residual standard error: 0.9821 on 126 degrees of freedom
## Multiple R-squared:  0.005247,   Adjusted R-squared:  -0.002648
## F-statistic: 0.6646 on 1 and 126 DF,  p-value: 0.4165
```

```
qqnorm(resid(m2.1))
```

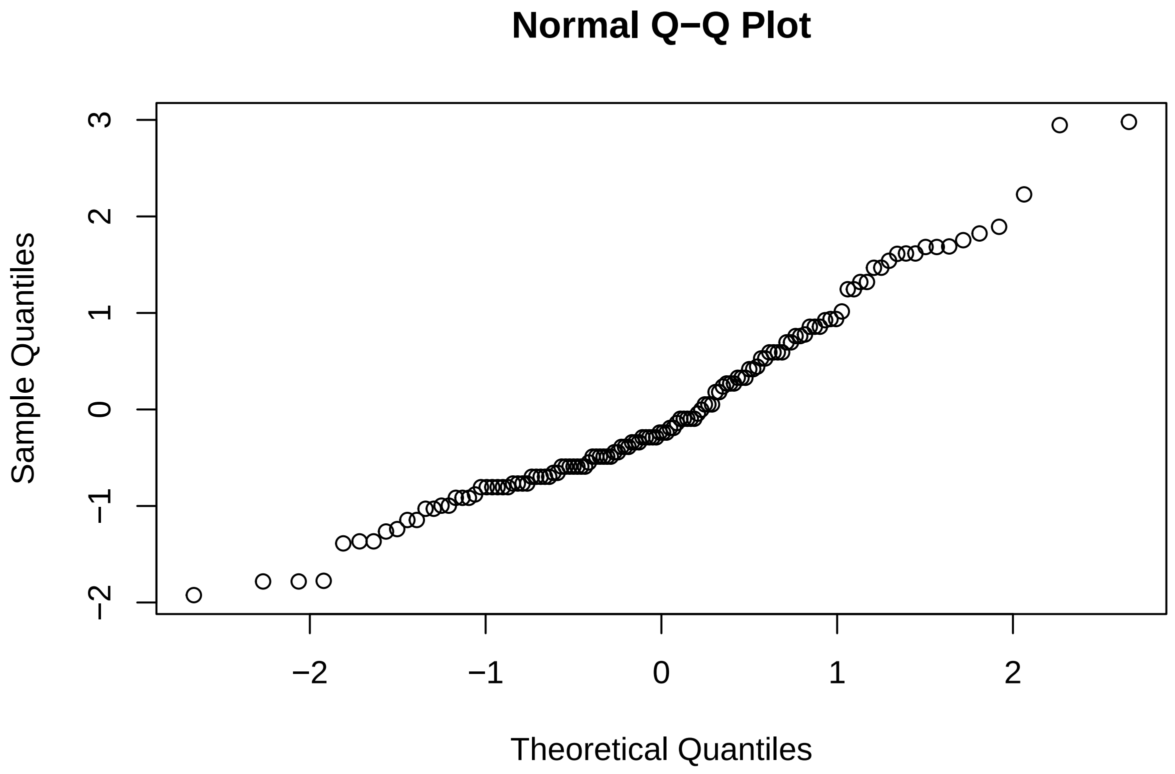


```
simoutbin500.2.1<-simulateResiduals(fittedModel=m2.1, n=250)
plot(simoutbin500.2.1)
```

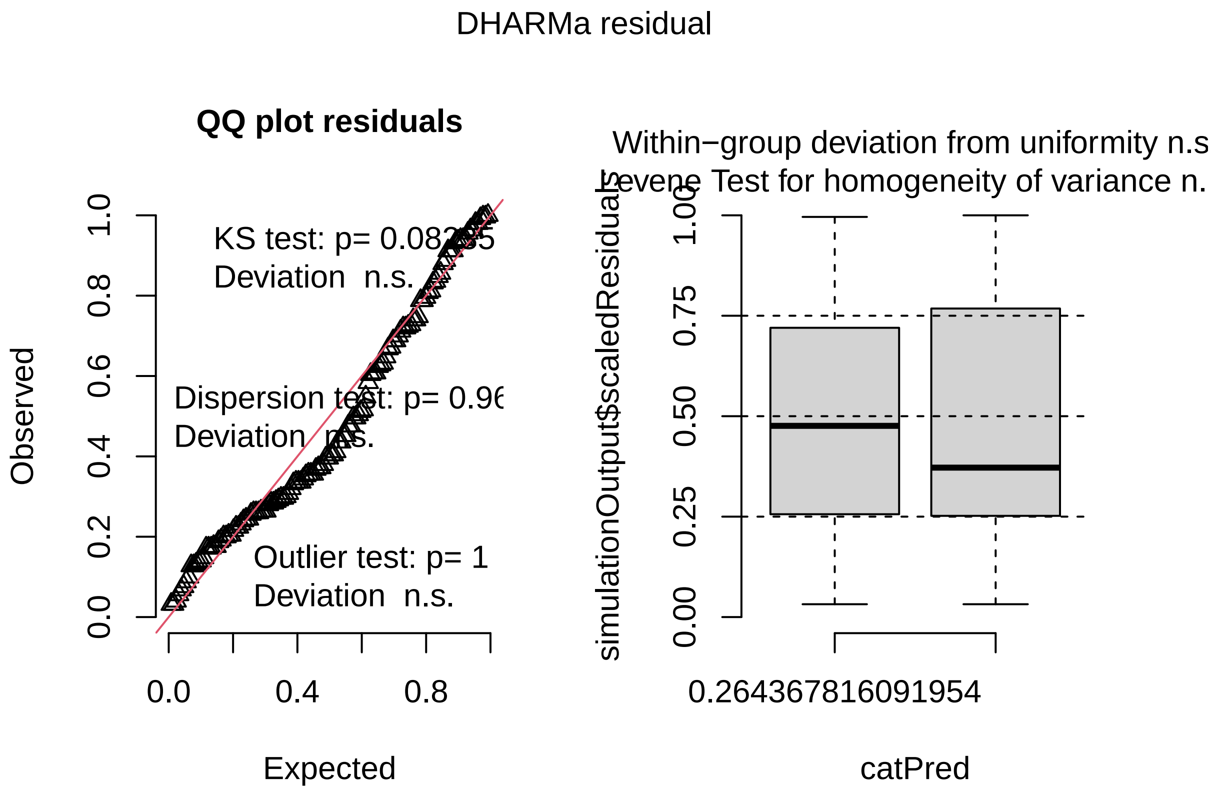

No model violations now, but worth keeping in mind that we removed the random effects.

Levels Load files Levels

```
K_W_lev <- read.table("alpha_div_levels.txt", header = T, fill=TRUE, row.names = 1, sep='\t')
AlDiv.df_lev <- as.data.frame(K_W_lev)
```

Data Spread Levels

Now I'm going to check whether the ASVs are normally distributed.

```
hist(AlDiv.df_lev$observed_otus)
```

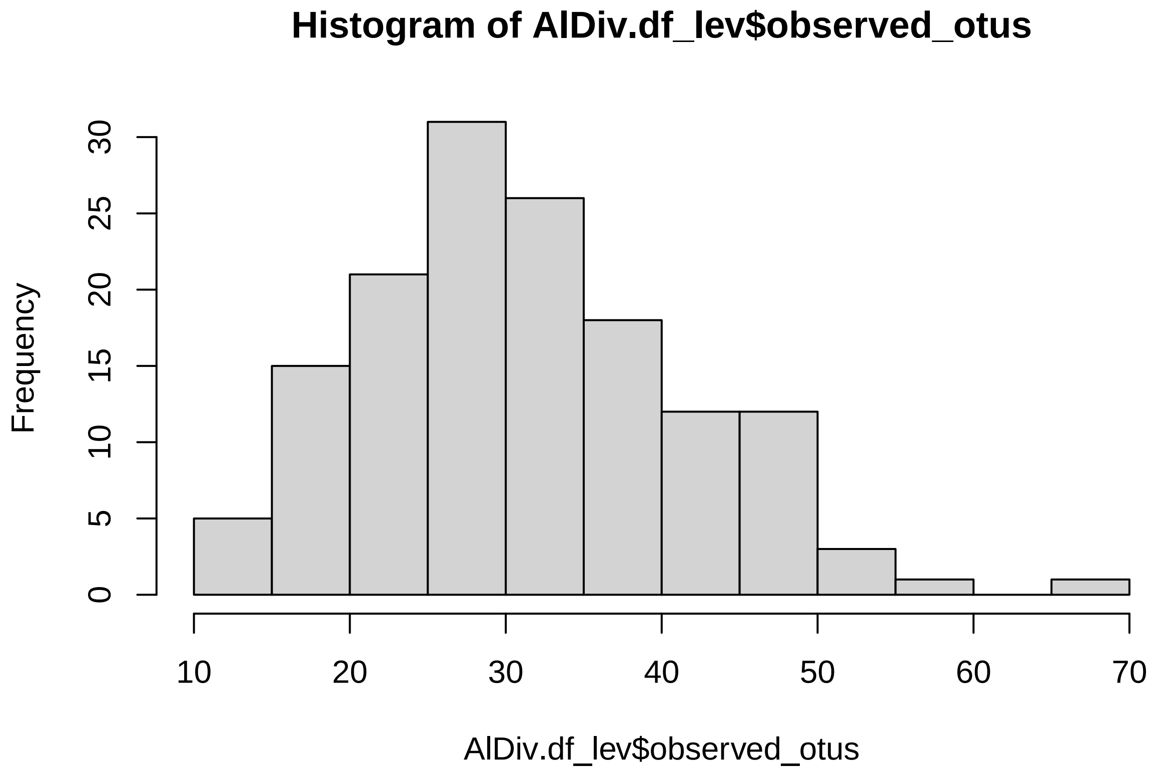

Looks good I don't have to transform this one, because downstream no model assumptions violated.

LMM model Levels

```
m3.0<-lmer(observed_otus ~ Code + (1|Colony) + (1|Cage_Num), data=AlDiv.df_lev, na.action="na.omit", REI
summary(m3.0)
```

```
## Linear mixed model fit by maximum likelihood  ['lmerMod']
## Formula: observed_otus ~ Code + (1 | Colony) + (1 | Cage_Num)
##    Data: AlDiv.df_lev
##
##      AIC      BIC   logLik deviance df.resid
##    695.0    707.9   -342.5    685.0       92
##
## Scaled residuals:
##     Min      1Q  Median      3Q     Max
## -1.9001 -0.6755  0.1038  0.5450  4.1996
##
## Random effects:
##  Groups   Name        Variance Std.Dev.
##  Cage_Num (Intercept) 12.48    3.533
##  Colony   (Intercept) 21.14    4.598
##  Residual             55.93    7.479
## Number of obs: 97, groups:  Cage_Num, 12; Colony, 9
##
## Fixed effects:
```

```
##              Estimate Std. Error t value
## (Intercept)  29.4842     2.9696   9.929
## Code          1.5390     0.8486   1.814
##
## Correlation of Fixed Effects:
##      (Intr)
## Code -0.684
```

```
Anova(m3.0)
```

```
## Analysis of Deviance Table (Type II Wald chisquare tests)
##
## Response: observed_otus
##      Chisq Df Pr(>Chisq)
## Code 3.289  1    0.06975 .
## ---
## Signif. codes:  0 '***' 0.001 '**' 0.01 '*' 0.05 '.' 0.1 ' ' 1
```

No significant effect.

```
plot(m3.0)
```

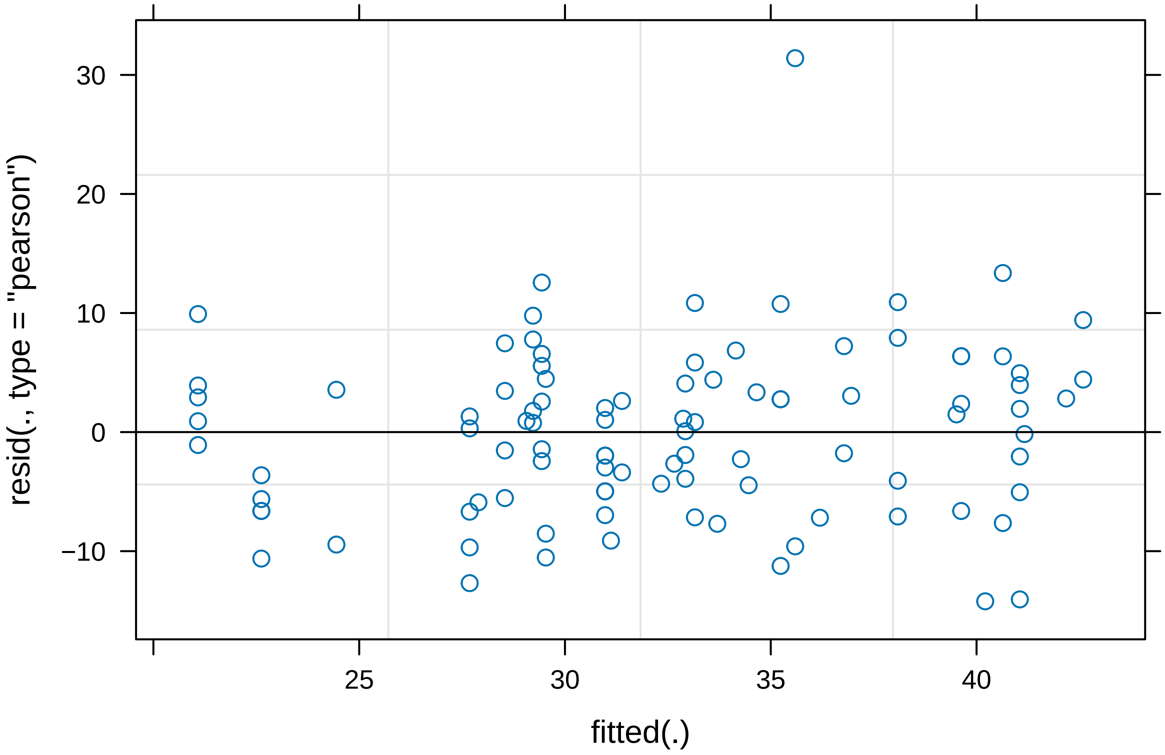


```
summary(m3.0)
```

```
## Linear mixed model fit by maximum likelihood  ['lmerMod']
## Formula: observed_otus ~ Code + (1 | Colony) + (1 | Cage_Num)
##    Data: AlDiv.df_lev
##
##      AIC      BIC   logLik deviance df.resid
##    695.0    707.9   -342.5    685.0       92
##
## Scaled residuals:
##     Min      1Q  Median      3Q     Max
## -1.9001 -0.6755  0.1038  0.5450  4.1996
##
## Random effects:
##  Groups   Name        Variance Std.Dev.
##  Cage_Num (Intercept) 12.48    3.533
##  Colony   (Intercept) 21.14    4.598
##  Residual             55.93    7.479
## Number of obs: 97, groups:  Cage_Num, 12; Colony, 9
##
## Fixed effects:
##             Estimate Std. Error t value
## (Intercept)  29.4842     2.9696   9.929
## Code          1.5390     0.8486   1.814
##
## Correlation of Fixed Effects:
##      (Intr)
## Code -0.684
```

```
qqnorm(resid(m3.0))
```

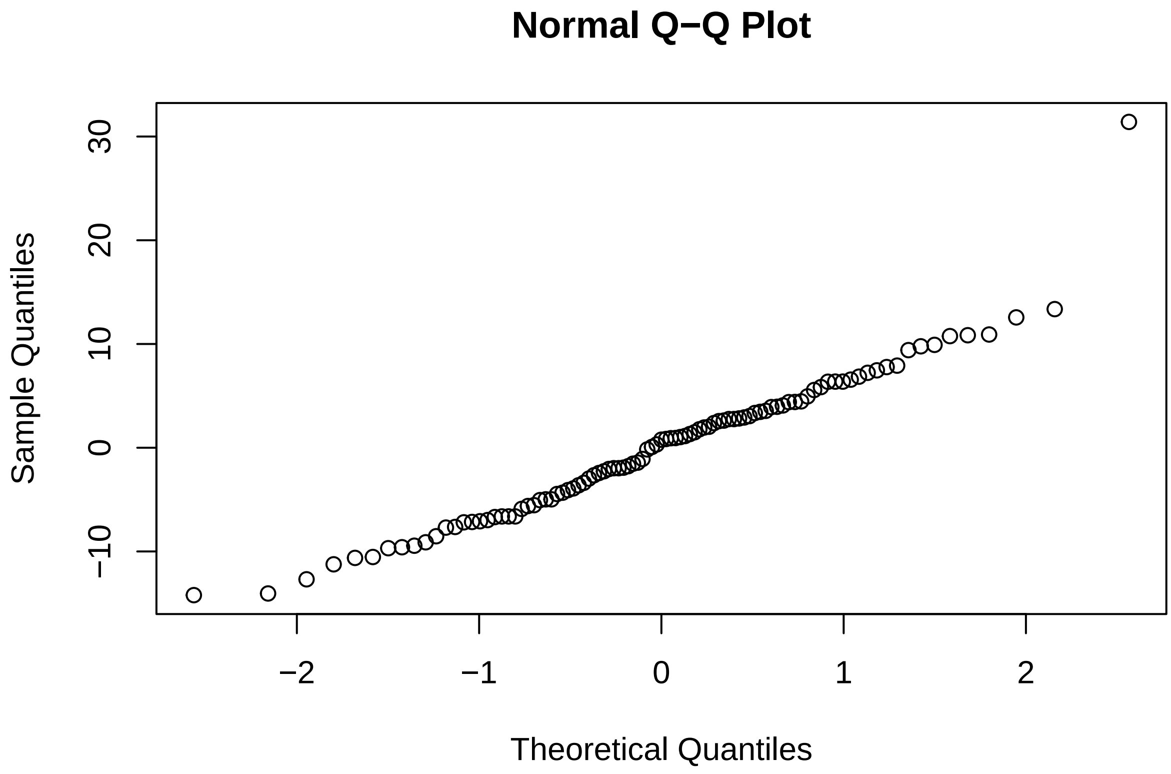


```
simoutbin500.3.0<-simulateResiduals(fittedModel=m3.0, n=250)
plot(simoutbin500.3.0)
```

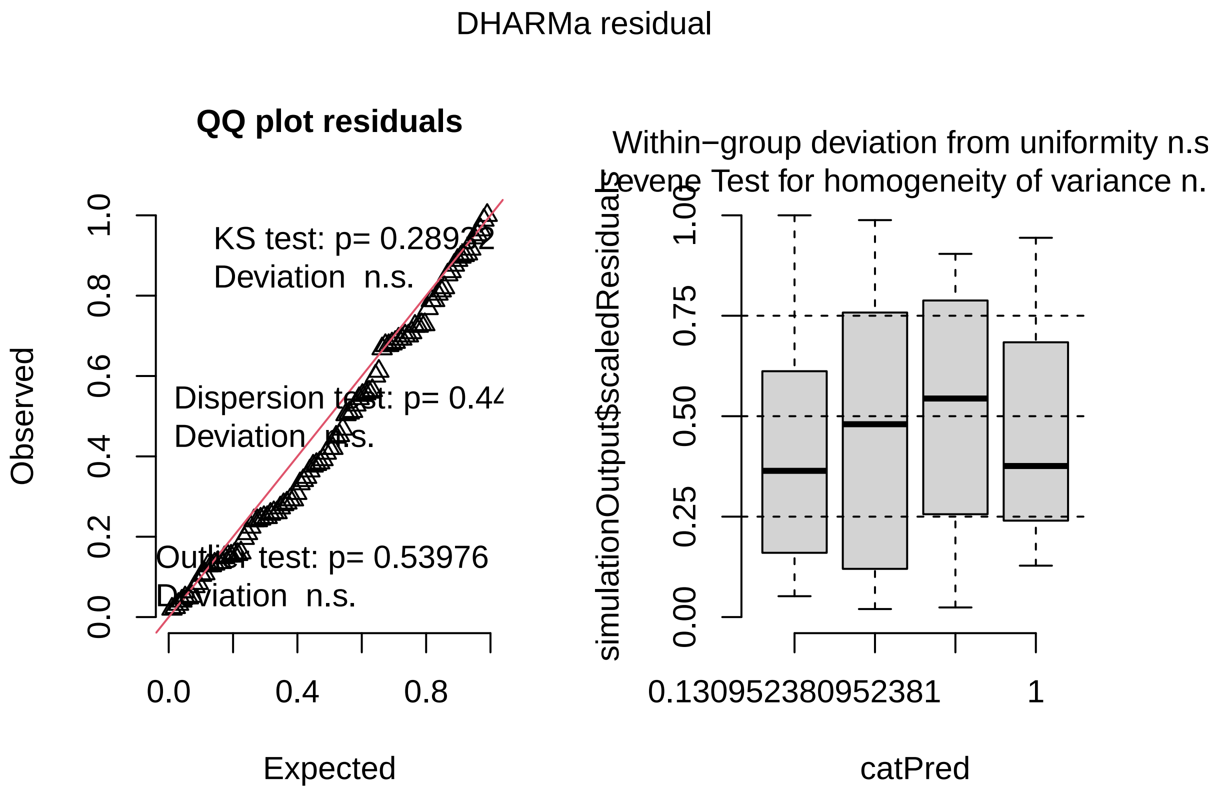

No model assumptions violated!

ANCOM

I ran these in qiime2 and there was some significant differential abundance. I'm going to just list the taxa that were diff. abundant for each analysis below. They are also in the supplemental files

Treatment Vs control 5 uncultured Lactobacillus, 1 Bombella, 1 uncultured Acetobacteraceae bacterium

Levels 3 uncultured gamma proteobacterium (Gilliamella) and one unknown bacteria

Resist 2 uncultured Lactobacillus sp.
