# Supplementary material for: Exploring the interactions between Nosema ceranae infection and the honey bee gut microbiome
Source: Sci Rep. 2024 Aug 29;14:20037. doi: 10.1038/s41598-024-67796-y (PMC11358482; doi:10.1038/s41598-024-67796-y)

**SUPPLEMENTAL INFORMATION**

**Table S1: Definitions of infection categories and mean infection levels of bees in these categories for all bees and for bees whose microbiomes were analyzed.** Experimental bees were each fed 40,000 freshly harvested *N. ceranae* spores in sucrose solution. Control bees received no spores but were sham treated (fed pure sucrose solution without spores). Spores were counted from gut dissections 12 days after feeding.

| **Bee type** | **Treatment** | **Infection category** | **Definition** | **Minimum spores** | **Maximum spores** |
| --- | --- | --- | --- | --- | --- |
| Bees whose | Experimental | Fed-spores-but-not-infected | No gut spores despite being fed live spores | 0 | 0 |
| microbiomes | Experimental | Low spore count | Spore counts < median | 1 | 1,822,500 |
| were | Experimental | Moderate spore count | Median to 75th quartile spore counts | 1,822,501 | 6,291,250 |
| analyzed | Experimental | High spore count | Spore counts > 75th quartile | 6,291,251 | 41,400,000 |
|  |  |  |  |  |  |
|  |  |  |  |  |  |
| **Bee type** | **Treatment** | **Infection category** | **N bees** | **Mean ± 1 SD** |  |
| All bees | Control | Control | 506 | 1,828 ± 8.347 |  |
|  | Experimental | Fed-spores-but-not-infected | 46 | 0 ± 0 |  |
|  | Experimental | Low spore count | 129 | 691,667 ± 611,650 |  |
|  | Experimental | Moderate spore count | 110 | 3,721,227 ± 1,353,601 |  |
|  | Experimental | High spore count | 211 | 15,821,706 ± 7,518,512 |  |
|  |  |  |  |  |  |
| **Bee type** | **Treatment** | **Infection category** | **N bees** |  |  |
| Bees whose | Control | Control | 93 | 0 ± 0 |  |
| microbiomes | Experimental | Fed-spores-but-not-infected | 47 | 0 ± 0 |  |
| were | Experimental | Low spore count | 30 | 613,667 ± 637,100 |  |
| analyzed | Experimental | Moderate spore count | 39 | 3,927,179 ± 1,361,359 |  |
|  | Experimental | High spore count | 38 | 13,387,500 ± 8,226,583 |  |

**Figure S1:** Rarefaction Curves. These are the rarefaction curves generated in QIIME2 by OTUs and samples. Based on these figures we determined 2,500 reads per sample would be an appropriate depth for further analyses.
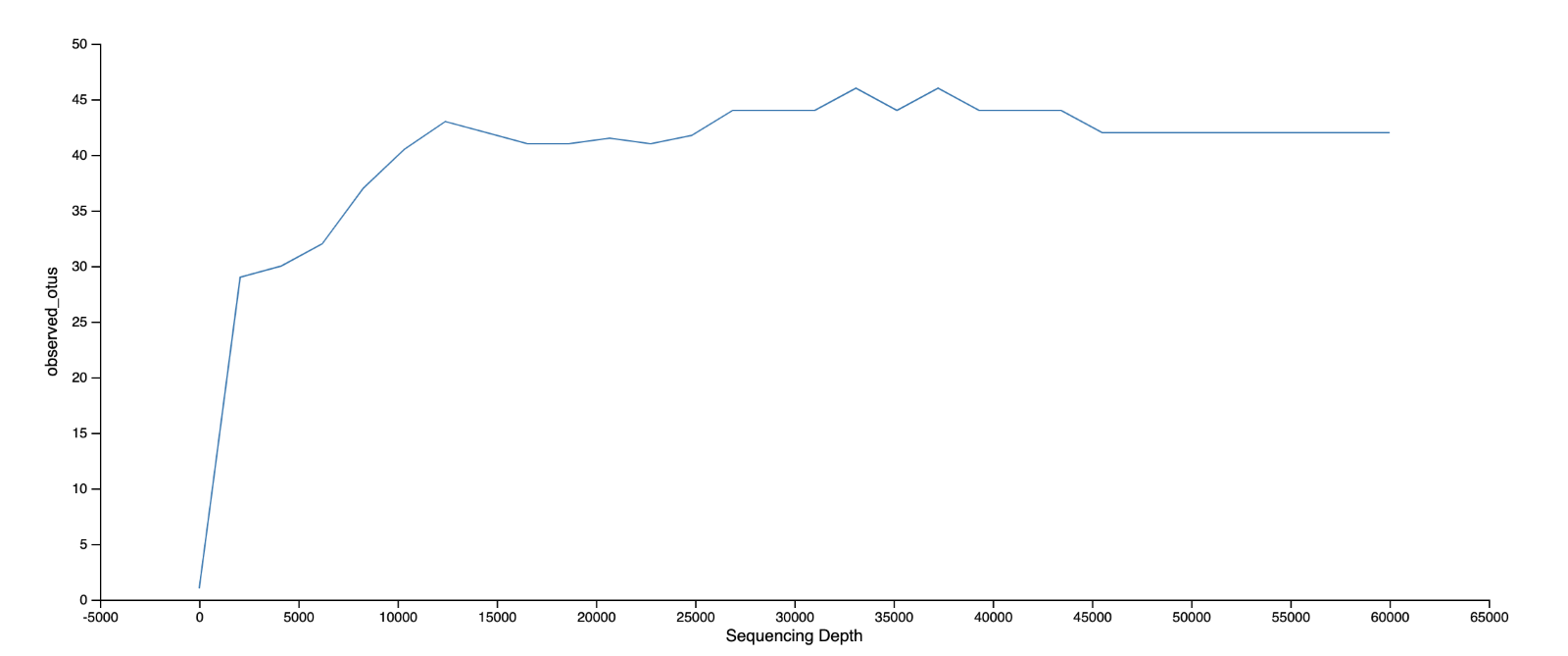


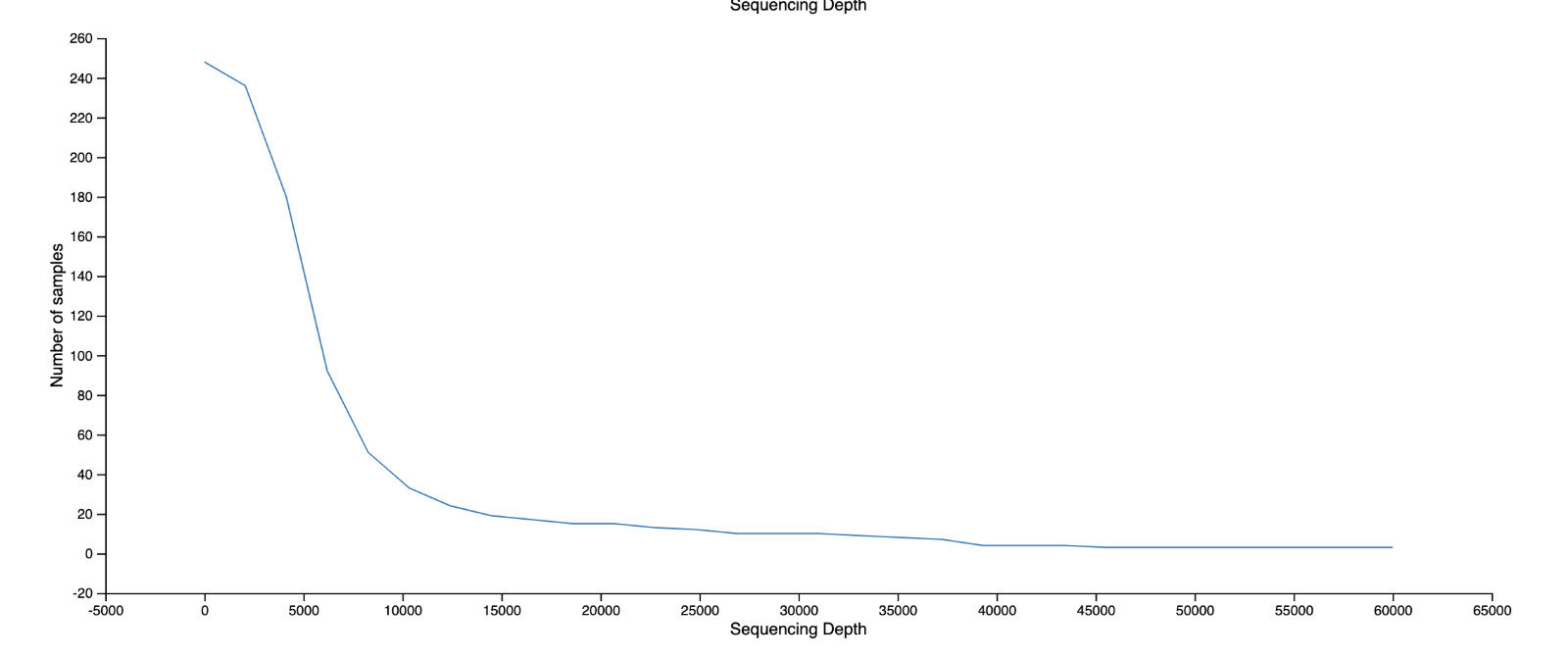


**Figure S2:** Bacterial Profiling. (A) The top eight most relatively abundant bacterial species based on infection level are shown (B) The top ten most relatively abundant bacterial genera based on infection level are shown.

A


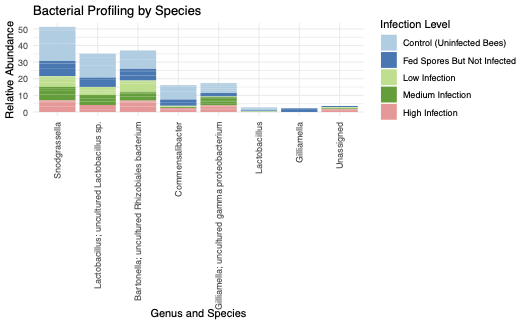


B


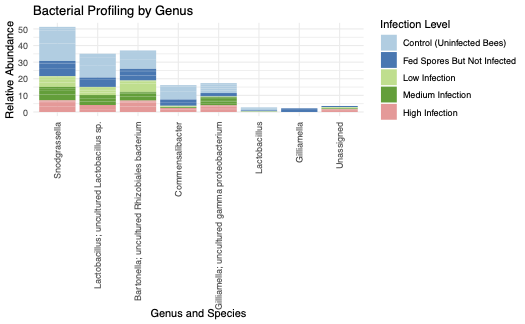

Supplement: Supplementary file 2 — Supplementary Information 2. [file 41598_2024_67796_MOESM2_ESM.docx]
